# cAAC‐Stabilized 9,10‐diboraanthracenes—Acenes with Open‐Shell Singlet Biradical Ground States

**DOI:** 10.1002/anie.202008206

**Published:** 2020-08-25

**Authors:** Christian Saalfrank, Felipe Fantuzzi, Thomas Kupfer, Benedikt Ritschel, Kai Hammond, Ivo Krummenacher, Rüdiger Bertermann, Raphael Wirthensohn, Maik Finze, Paul Schmid, Volker Engel, Bernd Engels, Holger Braunschweig

**Affiliations:** ^1^ Institut für Anorganische Chemie Julius-Maximilians-Universität Würzburg Am Hubland 97074 Würzburg Germany; ^2^ Institute for Sustainable Chemistry & Catalysis with Boron Julius-Maximilians-Universität Würzburg Am Hubland 97074 Würzburg Germany; ^3^ Institut für Physikalische und Theoretische Chemie Julius-Maximilians-Universität Würzburg Emil-Fischer-Strasse 42 97074 Würzburg Germany

**Keywords:** acenes, biradicals, bond Activation, boron, heterocycles

## Abstract

Narrow HOMO–LUMO gaps and high charge‐carrier mobilities make larger acenes potentially high‐efficient materials for organic electronic applications. The performance of such molecules was shown to significantly increase with increasing number of fused benzene rings. Bulk quantities, however, can only be obtained reliably for acenes up to heptacene. Theoretically, (oligo)acenes and (poly)acenes are predicted to have open‐shell singlet biradical and polyradical ground states, respectively, for which experimental evidence is still scarce. We have now been able to dramatically lower the HOMO–LUMO gap of acenes without the necessity of unfavorable elongation of their conjugated π system, by incorporating two boron atoms into the anthracene skeleton. Stabilizing the boron centers with cyclic (alkyl)(amino)carbenes gives neutral 9,10‐diboraanthracenes, which are shown to feature disjointed, open‐shell singlet biradical ground states.

## Graphical abstract

9,10‐diboraanthracenes (DBAs) stabilized by cyclic (alkyl)(amino)carbenes (cAACs) are prepared. EPR spectroscopy and quantum chemistry established disjointed singlet biradical ground states for these molecules, which provides experimental evidence for the predicted open‐shell configurations of larger acenes. The incorporation of two boron atoms is a suitable way to lower the HOMO–LUMO band gaps of acene‐type species.

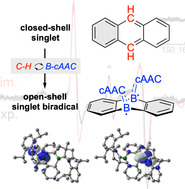

## Introduction

**Figure 1:**
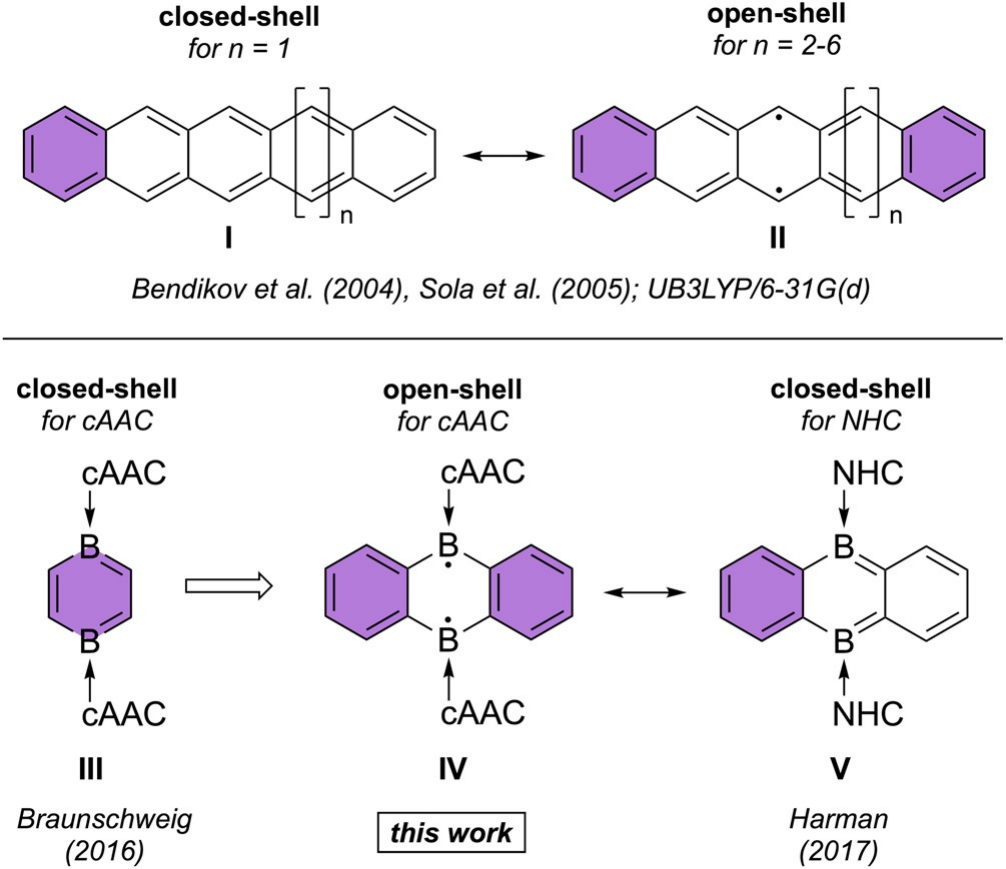
Closed‐shell and biradical resonance structures of (oligo)acenes (top; *n*=1–6) and neutral diboraacenes (bottom). Clar‐sextets are highlighted in purple. NHC=*N*‐heterocyclic carbene; cAAC=cyclic (alkyl)(amino)carbene.

Acenes are an important subclass of polycyclic aromatic hydrocarbons (PAHs) consisting of linearly fused benzene rings (Figure 1).^1^ Such molecules exhibit unique electronic properties that not only initiated a controversial theoretical debate,^2^ but also attracted attention of experimental and application‐oriented scientists.^1^ Theoretical studies predicted that (oligo)acenes from hexacene (*n*=2) to decacene (*n*=6) no longer possess closed‐shell configurations, but rather feature open‐shell singlet biradical ground states with disjointed character.^3^ This behavior was rationalized on the basis of decreasing HOMO–LUMO gaps and decreasing singlet‐triplet energy separations with increasing lengths of the conjugated π system. Thus, the energy required for breaking π bonds in the Kekulé representations of acenes (**I**) decreases as well, and can eventually be overcompensated for acenes with *n*=2–6 by adding open‐shell character to their ground states; the biradical resonance structure of acenes (**II**) shows an extra aromatic Clar‐sextet (Figure 1).2c We note that acenes larger than dodecacene were found to exhibit a singlet open‐shell polyradical character,^4^ and were predicted to behave as one‐dimensional organic conductors with a zero band gap.^2^, ^3^ Hence, with increasing number of fused benzene rings acenes gradually become organic p‐type semiconductors, which makes them highly relevant for organic electronic applications^1^, ^5^ such as field‐effect transistors,^6^ light‐emitting diodes,^7^ organic conductors,5b, 8 solar cells, and electronically pumped organic solid‐state injection lasers.^1^, ^5^ For instance, pentacene^9^ and hexacene^10^ were shown to possess unusually high charge‐carrier mobilities, making them attractive as efficient semi‐conducting materials in field‐effect transistors.

In addition, larger acenes share similarities to important carbonaceous materials such as graphene^11^ and carbon nanotubes,^12^ potentially facilitating applications such as spintronics,^13^ and plasmonics.^14^ However, the broad use of acenes larger than pentacene into such applications is still hampered by severe experimental difficulties in obtaining bulk quantities of pure materials due to increasing instability and insolubility. Valuable protocols for the bulk preparation of hexacene^10^ and heptacene^15^ have been developed only recently, and higher analogs (octacene, nonacene) could only be generated in cryogenic noble‐gas matrices so far.^16^ We note that despite these advances, the electronic structure of (oligo)acenes is still not completely understood; conclusive experimental evidence for the predicted significance of open‐shell contributions to the ground state of these molecules is still rare.

The implementation of boron atoms into organic molecules was also shown to be a quite efficient strategy for lowering their HOMO–LUMO gaps, mainly by raising the energy of the HOMO.^17^ We reasoned that this approach might also be viable for acenes, thus allowing the generation of open‐shell systems already for a smaller number of fused benzene rings. For this reason, we directed our research efforts towards the enlargement of the neutral, closed‐shell diborabenzene scaffold (**III**), introduced in 2016 by our group,^18^ to the 9,10‐diboraanthracene (DBA) skeleton (**IV**; Figure 1; cAAC=cyclic (alkyl)(amino) carbene). It should be noted that Harman's related NHC‐stabilized derivative **V** (NHC=*N*‐heterocyclic carbene, Figure 1) showed closed‐shell character,^19^ which suggested that further fine‐tuning of the acenes’ electronics is required to eventually prompt biradical formation, and to stabilize the resulting open‐shell configuration. Due to their unique σ donor and π acceptor capabilities, cAACs are powerful Lewis bases,^20^ which have already proven their efficiency in boron chemistry to support realization of uncommon and highly reactive, neutral molecules^21^ and radical species.^22^ For instance, diborenes of the type (L)(ER)B=B(ER)(L) (ER=SBu, SPh, SePh) feature a closed‐shell ground state for L=NHC, while a triplet ground state was found for L=cAAC; both compounds show distinct differences in their geometries.22j Beside higher steric demands of NHC ligands, differences in the nodal structures of HOMOs and LUMOs turned out to be important because they induce smaller gaps leading to considerably more stable triplet states in cAAC‐stabilized boron compounds.22k Thus, we were confident that cAAC‐stabilized 9,10‐DBAs **IV** (Figure 1) might be suitable candidates to make smaller acene analogs with open‐shell biradical ground states accessible. This assumption turned out to be true, and herein we report our results related to the synthesis, electronic structure and reactivity of two cAAC‐stabilized 9,10‐DBA derivatives. A combination of EPR spectroscopy and quantum chemistry studies was used to characterize the disjointed, open‐shell biradical singlet ground state of these molecules, which is expected to stimulate application‐oriented research efforts.

## Results and Discussion

**Figure 2:**
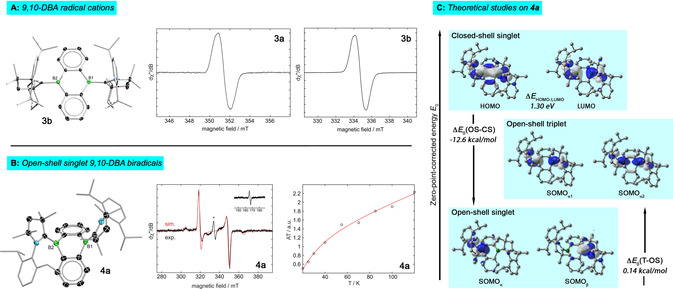
A) Molecular structure of radical cation **3 b** as its MgBr_3_^−^ salt in the solid state (left). Hydrogen atoms, (thf)MgBr_3_^−^ counter anion and some of the ellipsoids of the cAAC^Cy^ ligand have been omitted for clarity.^23^ Experimental CW X‐Band (9.85 GHz) EPR spectra of **3 a** (middle; *g=*2.0020) and **3 b** (right; *g=*2.0023) at room temperature. B) Molecular structure of biradical **4 a** in the solid state (left). Only one independent molecule of the asymmetric unit is shown. Hydrogen atoms and some of the ellipsoids of the cAAC^Me^ ligand have been omitted for clarity. Experimental CW X‐Band (9.85 GHz) EPR spectrum of **4 a** in frozen toluene solution at 20 K (middle). The inset shows the forbidden Δ*m*_S_=2 half‐field transition. The small center peak marked with an asterisk is due to a monoradical impurity. Key parameters for the simulation of the triplet state: *g*_1_=2.005, *g*_2_=2.003, *g*_3_=2.002, *D*=0.0284 cm^−1^, *E=*0.0003 cm^−1^. Representation of the temperature dependence of the double integral EPR intensity (*A*) of **4 a** in frozen toluene solution (right). Circles are the experimental results and the red line corresponds to the fit with the Bleaney–Bowers equation. C) Selected frontier molecular orbitals of **4 a** in its closed‐shell singlet (top), open‐shell triplet (middle), and open‐shell singlet configurations (bottom), and relevant adiabatic energy differences Δ*E*_0_ calculated at the UB3LYP‐D3(BJ)/def2‐SVP level of theory.

**Scheme 1:**
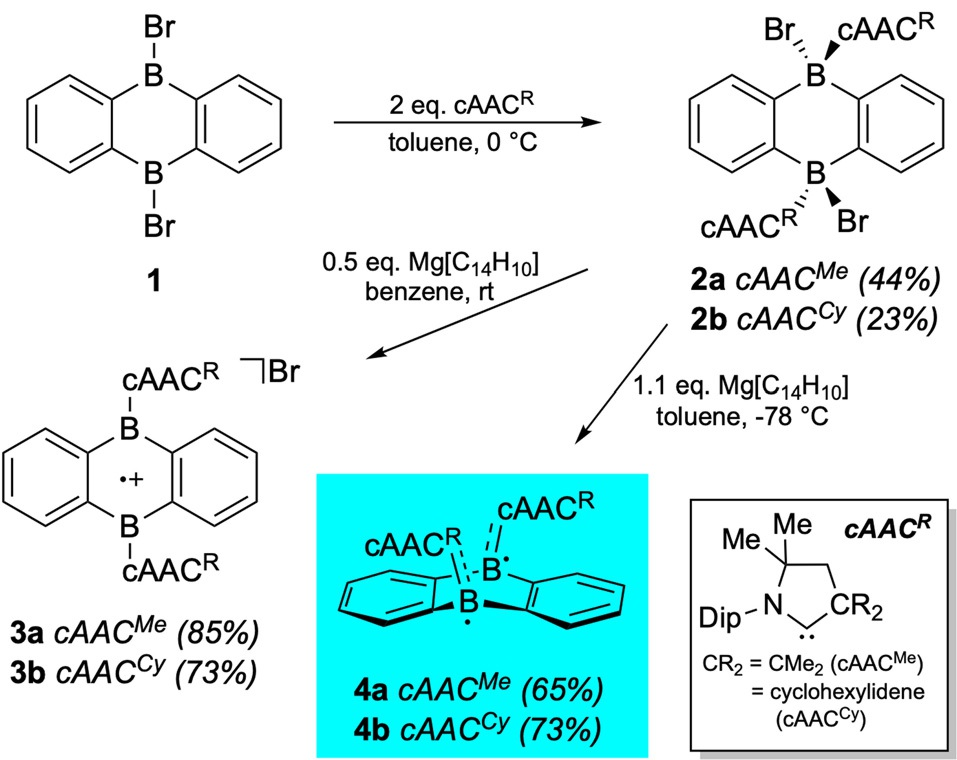
Synthesis of cAAC^R^‐stabilized, monoradicals **3 a**/**3 b**, and open‐shell singlet biradicals **4 a**/**4 b**.

To this end, we reacted literature‐known DBA precursor **1** with two equivalents of a cAAC^R^ Lewis base to afford *trans*‐configured bis(adducts) **2 a** (cAAC^Me^) and **2 b** (cAAC^Cy^) in moderate isolated yields as off‐white solids (Scheme 1). Their identity was clearly verified by solid‐state NMR spectroscopy, which provided ^11^B NMR signals with typical chemical shifts for the tetracoordinate boron centers (**2 a** *δ*=−6.1 ppm; **2 b** *δ*=−4.3 ppm), and elemental analysis. Both adducts were found unexpectedly labile in solution, readily decomposing into undefined species at room temperature within hours, which precluded their structural characterization in the solid state. The reason for this behavior is not known so far. However, subsequent treatment of **2 a**/**2 b** with 0.5 equivalents of Bogdanovic magnesium, [Mg(thf)_3_][C_14_H_10_], resulted in their selective one‐electron reduction and formation of highly‐colored 9,10‐DBA radical cations **3 a** and **3 b**, respectively (Scheme 1). Solutions of deep‐green **3 a**/**3 b** are NMR silent, but exhibit broad singlets centered around *g=*2.00 (**3 a** *g=*2.0020; **3 b** *g=*2.0023) in their CW X‐band (9.85 GHz) EPR spectra in 1,2‐difluorobenzene at room temperature (Figure 2 A). The absence of any hyperfine coupling interactions suggests strong delocalization of the unpaired electron of **3 a**/**3 b**, and hence symmetric, ionized structures similar to the Lewis depiction used in Scheme 1. This was further validated by an X‐ray diffraction study on cAAC^Cy^‐substituted radical cation **3 b**, which was crystallized as its MgBr_3_^−^ salt (Figure 2 A).^23^ Thus, **3 b** exhibits an essentially planar 9,10‐DBA skeleton with tricoordinate boron atoms in the solid state. In line with its delocalized nature, the central B_2_C_4_ core of **3 b** features equalized endocyclic B−C_DBA_ bonds with distances (1.538(5)–1.548(5) Å) implying B−C multiple bond character. Exocyclic B−C_cAAC_ distances (1.632(5), 1.638(5) Å) are consistent with typical dative bonding interactions.21a, 22c, 24 We note that the related NHC‐stabilized radical cation studied by Harman and co‐workers showed similar structural (B−C_DBA_ 1.523–1.536 Å; B−C_cAAC_ 1.602, 1.605 Å) and spectroscopic (*g=*2.00) parameters.^19^

Two‐electron reduction of precursors **2 a**/**2 b** employing 1.1 equiv. of [Mg(thf)_3_][C_14_H_10_] yielded NMR‐silent, orange‐colored solutions, from which compounds **4 a** and **4 b** were isolated in yields of 65 % and 73 %, respectively (Scheme 1). Temperature‐dependent EPR studies in toluene solutions revealed the presence of two unpaired electrons, and hence, the formation of biradical 9,10‐DBAs with open‐shell triplet configurations. This is in stark contrast to the observations made by the Harman group, which obtained planar π‐conjugated, closed‐shell 9,10‐DBA systems (*cf*. **V**, Figure 1).^19^ Thus, the usage of cAACs as stabilizing Lewis bases clearly met our initial expectations in terms of favoring/stabilizing the biradical resonance structure of smaller acenes. In fact, the temperature dependence of the double integral EPR intensities showed that our molecules **4 a**/**4 b** possess singlet ground states (Figure 2 B). Fitting of the EPR data with the Bleaney–Bowers equation provided very small singlet‐triplet energy gaps of Δ*E*(T‐S)=0.10 kcal mol^−1^ (**4 a**) and 2.43 kcal mol^−1^ (**4 b**), which is consistent with a significant population of the triplet state at room temperature. These findings agree very well with the results of our theoretical calculations using the unrestricted broken symmetry B3LYP method (UB3LYP‐D3(BJ)/def2‐SVP) that lead to triplet states of **4 a**/**4 b** marginally higher in energy than their open‐shell singlet solutions. Adiabatic (Δ*E*_0_(T‐OS)) and vertical (Δ*E*(T‐OS)) singlet‐triplet gaps were calculated to be Δ*E*_0_(T‐OS)=0.14 (**4 a**), 0.15 (**4 b**) kcal mol^−1^ and Δ*E*(T‐OS)=0.11 (**4 a**), 0.13 (**4 b**) kcal mol^−1^, respectively; thus they are considerably smaller than those determined theoretically for (oligo)acenes (*n*=2–6; Δ*E*(T‐OS)=5.7–10.3 kcal mol^−1^; UB3LYP/6‐31G(d)).^3^ However, our quantum chemistry studies verified that the open‐shell singlet biradical ground states of **4 a**/**4 b** are of disjointed character (Figure 2 C), as predicted for (oligo)acenes.^3^ These conclusions are also supported by high‐level *ab‐initio* calculations (CASSCF/NEVPT2) on **4 a**.^25^ Here, the RI‐NEVPT2/def2‐SVP treatment of **4 a** using reference CASSCF wave functions with (2,2) and (6,6) active spaces verified that the open‐shell singlet state is more stable than the triplet by 0.05 and 0.07 kcal mol^−1^, respectively. We note that EPR studies on **4 b** were hampered by its lability in solution, and the formation of a monoradical decomposition product. The EPR spectrum after complete decomposition of **4 b** is highly reminiscent of a cAAC^Cy^‐stabilized boryl radical (*g*_iso_=2.0024, *a*(B)=2.9 MHz; *a*(N)=16 MHz)^23^ and is shown in the Supporting Information (Figure S11). DFT optimization of **4 a**/**4 b** in their hypothetical closed‐shell singlet configurations further revealed that these solutions are significantly higher in energy than the open‐shell singlet biradical ground states (Δ*E*_0_(OS‐CS)=−12.59 (**4 a**), −12.26 (**4 b**) kcal mol^−1^; Δ*E*(OS‐CS)=−17.21 (**4 a**), −16.79 (**4 b**) kcal mol^−1^). Moreover, the high efficiency of our boron implementation approach for mimicking the electronics of acenes with a higher number of fused benzene rings becomes clearly evident when comparing the HOMO–LUMO gaps. The calculated HOMO–LUMO gaps of **4 a** and **4 b** (Δ*E*_HOMO–LUMO_=1.30 (**4 a**), 1.32 (**4 b**) eV) in their hypothetical closed‐shell configurations (B3LYP‐D3(BJ)/def2‐SVP) are very small; in fact, these values are dramatically smaller than that of the carbon analog anthracene (Δ*E*_HOMO–LUMO_=3.35 eV),4a, 26 and even smaller than those found in (oligo)acenes up to nonacene (Δ*E*_HOMO–LUMO_=1.43 eV).^16^

Similar to our observations made for diborenes (L)(ER)B=B(ER)(L),22j, 22k transition from a closed‐shell electronic configuration in **V** (L=NHC) to open‐shell ground states in **4 a**/**4 b** upon changing the Lewis base to cAAC^R^ comes along with major changes of the diboraanthracene geometry in the solid‐state structures of biradicals **4 a**/**4 b** (Figures 2 B, S18).^23^ Thus, **4 a** and **4 b** feature strongly bent DBA skeletons with acute angles between the planes of the two fused benzene rings (**4 a** 68.4°, 69.2°; **4 b** 73.0°). The coordination sphere of the boron atoms is close to trigonal‐planar (**4 a** *Σ*_B1‐4_ each 359.4°; **4 b** *Σ*_B_=359.3, 359.7°); however, they are significantly bent out of the central B_2_C_4_ ring plane by 44.0–45.3°. Such bent, butterfly‐type structures were also reported for related isoelectronic, quinoidal anthracenes with carbon‐based substituents in 9,10‐positions.^27^ The strong electronic impact of the cAAC^R^ ligands becomes evident by inspection of the exocyclic B−C_cAAC_ bonds of **4 a**/**4 b** (1.506(7)–1.531(2) Å). These are notably shortened as compared to radical cations **3 a**/**3 b** with typical dative single bonds (1.632(5), 1.638(5) Å), which implies substantial delocalization of the two unpaired electrons in **4 a**/**4 b** and B−C_cAAC_ multiple bond character. Simultaneously, endocyclic B−C_DBA_ bonds (1.569(8)–1.595(7) Å) experience distinct elongation upon biradical formation (*cf*. **3 a**/**3 b** 1.538(5)–1.548(5) Å). As a side note, it is worth mentioning that biradicals **4 a**/**4 b** can also be accessed by one‐electron reduction of radical cations **3 a**/**3 b** using 0.5 equivalents of Bogdanovic magnesium, even though this is not the preferred method.

**Figure 3:**
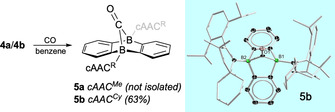
Reactions of biradicals **4 a**/**4 b** with CO to afford bicyclic molecules **5** (left). Molecular structure of **5 b** in the solid state (right).^23^ Hydrogen atoms and some of the ellipsoids of the cAAC^Cy^ ligands have been omitted for clarity.

Earlier work has demonstrated the ability of the central B_2_C_4_ ring of neutral and dianionic 9,10‐DBAs to undergo cycloaddition reactions with unsaturated organic substrates such as ethylene, and to cooperatively activate small molecules such as O_2_, CO_2_, and alkynes.^19^, ^28^ For this reason, we targeted the reactivity of biradicals **4 a**/**4 b** exemplarily towards gaseous CO (Figure 3). Thus, exposure of degassed solutions of **4 a**/**4 b** in benzene to one atmosphere of CO resulted in the gradual fixation of CO, eventually converting **4 a**/**4 b** into bicyclic closed‐shell molecules **5 a** and **5 b**, in which the two boron centers of the B_2_C_4_ core are now tetracoordinate (**5 a** *δ*_11B_=−0.4 ppm; **5 b** *δ*_11B_=−2.0 ppm) and bridged by a carbonyl entity. Single‐crystal X‐ray diffraction served to verify the structure of **5 b** as possessing two bridgehead boron atoms and an exocyclic C=O group (Figure 3).^23^ As expected, B−C_DBA_ (1.609(3)–1.649(3) Å) and B−C_cAAC_ (1.589(3), 1.630(3) Å) bond lengths show values that are commonly associated with covalent^29^ and dative21a, 22c, 24 single bond interactions between these atoms, respectively. By contrast, B−C_CO_ bonds involving the CO bridge are notably longer (1.695(3), 1.755(3) Å), while the C=O separation distance (1.205(2) Å) indicates double bond character.^30^ Even though cAAC^Me^‐based biradical **4 a** also reacted with CO to afford bicyclic product **5 a**, the reaction proceeded less selectively than for **4 b**, and we were not able to separate it analytically pure from its reaction mixtures. We note that such a reactivity is not without precedent in boron chemistry, and has been described for example, for B−B multiple bond systems such as diborenes and diborynes.22j, 31 Nonetheless, the reactions with CO clearly demonstrate the high potential of 9,10‐DBAs to follow rather uncommon reactivity pathways.

## Conclusion

In summary, we could show that the implementation of boron atoms is a powerful approach to efficiently lower the HOMO–LUMO gap of acene‐type molecules. With ancillary support by cyclic (alkyl)(amino)carbenes, diboraanthracenes are thus capable of making the theoretically predicted open‐shell ground state of (oligo)acenes (hexacene to decacene) accessible and energetically favorable already for a smaller number of fused benzene rings, that is, upon going from (closed‐shell) 1,4‐diborabenzenes (**III**) to 9,10‐diboraanthracenes (**IV**). Our results have clearly verified disjointed, open‐shell singlet biradical ground states for cAAC‐stabilized 9,10‐diboraanthracenes **4 a** and **4 b** (EPR, X‐ray, QM). Further exploiting this methodology might help to overcome many of the experimental issues associated with the synthesis of larger acenes, eventually establishing diboron analogs of acenes as alternative and highly efficient materials for organic electronic applications. Future work in our group will focus on the realization of larger diboron‐based acene molecules such as 9,10‐diborapentacene, which is expected to be readily accessible via slight modification of the current synthetic protocols, and in particular, on a detailed investigation of the electronics and application spectrum of these uncommon boron species.

## Conflict of interest

The authors declare no conflict of interest.

## Supporting information

As a service to our authors and readers, this journal provides supporting information supplied by the authors. Such materials are peer reviewed and may be re‐organized for online delivery, but are not copy‐edited or typeset. Technical support issues arising from supporting information (other than missing files) should be addressed to the authors.

Supplementary
