# Supplementary material for: cAAC‐Stabilized 9,10‐diboraanthracenes—Acenes with Open‐Shell Singlet Biradical Ground States
Source: Angew Chem Int Ed Engl. 2020 Aug 25;59(43):19338–43. doi: 10.1002/anie.202008206 (PMC7589216; doi:10.1002/anie.202008206)
Supplement: Supplementary file 1 — Supplementary [file ANIE-59-19338-s001.pdf]

Angewandte Chemie
Eine Zeitschrift der Gesellschaft Deutscher Chemiker

Supporting Information

cAAC-Stabilized 9,10-diboraanthracenes—Acenes with Open-Shell Singlet Biradical Ground States

*Christian Saalfrank, Felipe Fantuzzi, Thomas Kupfer, Benedikt Ritschel, Kai Hammond, Ivo Krummenacher, Rüdiger Bertermann, Raphael Wirthensohn, Maik Finze, Paul Schmid, Volker Engel, Bernd Engels, and Holger Braunschweig**

anie_202008206_sm_miscellaneous_information.pdf

Supporting Information

This PDF file includes:

Materials and Methods
Figs. S1 to S24
Tables S1 to S3
Additional References

S1 Synthetic details and characterization of compounds

General experimental considerations: All reactions were carried out under an atmosphere of dry argon using standard Schlenk line and glovebox techniques. NMR spectra were obtained from *Bruker Avance 400* and *500* NMR spectrometers at room temperature. Chemical shifts (δ) are given in ppm and are internally referenced to the carbon nuclei ($^{13}C\{^{1}H\}$) or residual protons (^{1}H) of the solvent. NMR spectra were referenced to $SiMe_4$ (^{1}H, ^{13}C), or $BF_3{\cdot}OEt_2$ (^{11}B) as external standards Solid-state NMR-data were obtained with a *Bruker DSX 400* NMR spectrometer. Microanalyses (C, H, N) were performed on an *Elementar vario MICRO cube* elemental analyzer. HRMS were measured on an *Exactive Plus Orbitrap-HRMS* manufactured by *Thermo Scientific*. UV/VIS-measurements were performed at *JASCO-V660* and *Mettler Toledo* UV spectrometers. Absorptions maxima are given in nm with shoulders in brackets, the global maximum being underlined. EPR measurements at X-band (9.4 GHz) were carried out using a *Bruker ELEXSYS E580 CW EPR spectrometer* equipped with an *Oxford Instruments helium cryostat (ESR900)* and a *MercuryiTC* temperature controller. The spectral simulations were performed using *MATLAB 9.6 (2019a)* and the *EasySpin 5.2.25* toolbox.[1] C_6D_6 was purchased from Sigma Aldrich, degassed by three freeze-pump-thaw cycles, and dried over molecular sieves. Other solvents were dried by storage over, and distillation from sodium (benzene, toluene), Na/K alloy (hexanes, pentane, THF), 4Å molecular sieves (1,2-difluorobenzene) under an argon atmosphere. Solvents were stored under argon over activated 4 Å molecular sieves. cAAC^{Me},[2] cAAC^{Cy},[2] 9,10 Dibromo-9,10-dihydro-9,10-diboraanthracene,[3] and Bogdanovic magnesium, $[Mg(thf)_3][C_{14}H_{10}]$,[4] were prepared according to literature methods. S_8 and CO (Grade 5.0) were purchased from Sigma-Aldrich and Linde, respectively, and used as received.

Synthesis and characterization of 2a

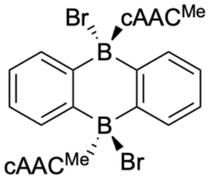

A solution of 9,10-dibromo-9,10-dihydro-9,10-diboraanthracene (1.62 g, 4.86 mmol) in toluene (10 mL) was cooled to –78 °C and reacted dropwise with a solution of $cAAC^{Me}$ (2.77 g, 9.72 mmol) in toluene (5 mL). The mixture was allowed to reach 0 °C, and stirred at that temperature for 14 h, during which time a white precipitate formed. The solvent was reduced to appoximately half of the volume *in vacuo*. The off-white solid was collected on a medium porosity frit, washed with toluene and hexanes (3 x 5 mL each), and dried *in vacuo*. Yield: 1.93 g (2.14 mmol, 44%) of an off-white solid. Note: Due to the low solubility and high lability of **2a**, no reliable solution NMR parameter could be obtained. Also, no solid-state ^{1}H NMR data are available.

^{13}C NMR (VACP/MAS, 13.5 kHz): δ = 233.3 ($C_{carbene}$), 210.1 ($C_{q\text{-aryl}}$), 193.7 ($C_{q\text{-aryl}}$), 149.3 ($C_{i\text{-aryl}}$), 145.8 ($C_{o\text{-aryl}}$), 144.1 ($C_{o\text{-aryl}}$), 137.5 ($C_{m\text{-aryl}}$), 136.2 ($C_{m\text{-aryl}}$), 130.9 ($C_{p\text{-aryl}}$), 128.9 ($C_{anthracene}$), 124.5 ($C_{anthracene}$), 84.8 ($C(CH_3)_2$), 83.3 ($C(CH_3)_2$), 56.6 (CH_2), 53.2 (CH_2), 34.9 (CH_3), 30.4 (CH_3), 29.1 (CH_3), 26.7 (CH_3).

^{11}B NMR (VACP/MAS, 14.8 kHz): δ = –6.1.

Elemental analysis calculated for $C_{72}H_{103}B_2N_3Br_4$ ($(cAAC^{Me})_2DBA(cAAC^{Me})(H_2Br_2)$): C 63.97, H 7.68, N 3.11; found: C 63.95, H 7.69, N 3.12.

Synthesis and characterization of 2b

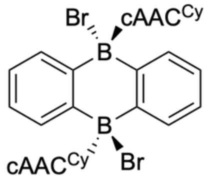

A solution of 9,10-dibromo-9,10-dihydro-9,10-diboraanthracene (1.62 g, 4.86 mmol) in toluene (10 mL) was cooled to –78 °C and reacted dropwise with a solution of $cAAC^{Cy}$ (6.32 g, 19.4 mmol) in toluene (20 mL). The mixture was allowed to reach 0 °C, and stirred at that temperature for 14 h, during which time a white precipitate formed. The solvent was reduced to appoximately half of the volume *in vacuo*. The off-white solid was collected on a medium porosity frit, washed with toluene and hexanes (3 x 5 mL each), and dried *in vacuo*. Yield: 1.10 g (1.12 mmol, 23%) of an off-white solid. Note: Due to the low solubility and high lability of **2b**, no reliable solution NMR parameter could be obtained. Also, no solid-state ^{1}H NMR data are available.

^{13}C NMR (VACP/MAS, 13.5 kHz): δ = 231.2 ($C_{carbene}$), 206.2 ($C_{q\text{-aryl}}$), 192.7 ($C_{q\text{-aryl}}$), 151.5 ($C_{i\text{-aryl}}$), 144.6 ($C_{o\text{-aryl}}$), 143.5 ($C_{o\text{-aryl}}$), 138.1 ($C_{m\text{-aryl}}$), 135.6 ($C_{m\text{-aryl}}$), 133.3 ($C_{p\text{-aryl}}$), 129.4 ($C_{p\text{-aryl}}$), 125.3 ($C_{anthracene}$), 121.6 ($C_{anthracene}$), 81.6 ($C(CH_3)_2$), 61.6 (R_2C_{Cy}), 44.6 (CH_2), 37.3 (CH_2), 31.2 ($C_{iPr}(CH_3)_2$), 29.3 ($C(CH_3)_2$), 26.1 (CH_2), 23.2(CH_2).

^{11}B NMR (VACP/MAS, 14.8 kHz): δ = –4.3.

Elemental analysis calculated for $C_{58}H_{78}B_2N_2Br_2(HBr)$: C 65.37, H 7.47, N 2.63; found: C 65.39, H 7.48, N 2.63.

Synthesis and characterization of 3a

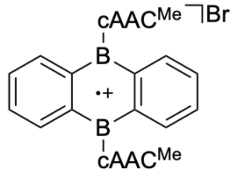

A suspension of **2a** (200 mg, 221 μmol) in benzene (3 mL) was reacted with [Mg(thf)$_3$][$C_{14}H_{10}$] (46.3 mg, 111 μmol) at room temperature. The mixture was and stirred for 4 h, after which time 2/3 of the solvent were removed under reduced pressure. The mixture was filtrated, and the residue was washed with hexanes (5 x 5 mL), and dried *in vacuo*. The solid thus obtained was extracted into a minimum amount of 1,2-difluorobenzene, filtered over a medium porosity frit, and dried *in vacuo*. Yield: 154 mg (188 μmol, 85%) of a green solid that proved NMR silent.

UV/vis (1,2-$C_6H_4F_2$): λ_{max} = <u>321</u>, 403, 471(446).

FTMS/pESI calculated for $C_{52}H_{70}B_2BrN_2$: m/z = 823.49; found: m/z = 823.49.

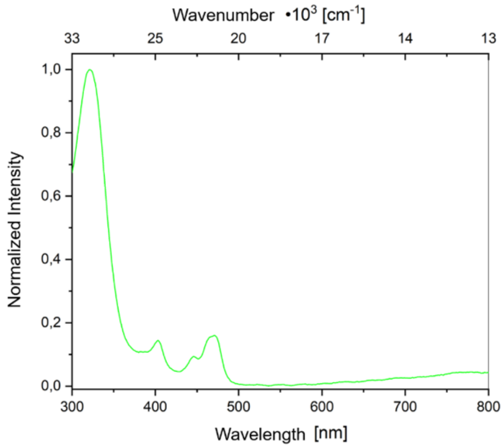

Fig. S1 | UV/vis spectrum of 3a in 1,2-$C_6H_4F_2$.

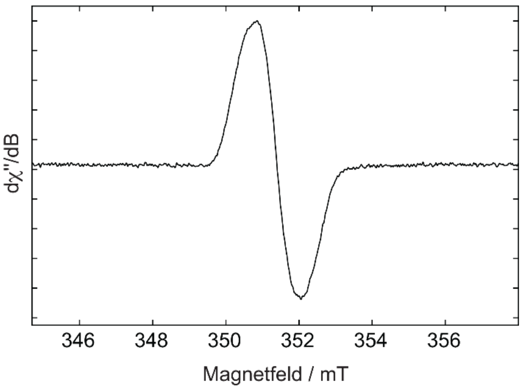

Fig. S2 | CW X-Band (9.85 GHz) EPR-spectrum of 3a at room temperature (*g* = 2.0020).

Synthesis and characterization of 3b

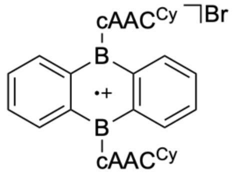

A suspension of **2b** (40.0 mg, 40.6 μmol) in benzene (1 mL) was reacted with $[Mg(thf)_3][C_{14}H_{10}]$ (8.5 mg, 20.3 μmol) at room temperature. The mixture was and stirred for 4 h, after which time 2/3 of the solvent were removed under reduced pressure. The mixture was filtrated, and the residue was washed with hexanes (5 x 1 mL) and dried *in vacuo*. The solid thus obtained was extracted into a minimum amount of 1,2-difluorobenzene, filtered over a medium porosity frit, and dried *in vacuo*. Crystals suitable for X-ray diffraction were obtained by slow evaporation of solutions of **3b** in 1,2-difluorobenzene into naphthalene. Yield: 26.8 mg (29.7 μmol, 73%) of a green solid that proved NMR silent.

UV/vis (1,2-$C_6H_4F_2$): λ_{max} = 364, 447, <u>460</u>, 479, 773(695).

HRMS/LIFDI calculated for $C_{58}H_{78}B_2BrN_2$: m/z = 903.55; found: m/z = 903.55.

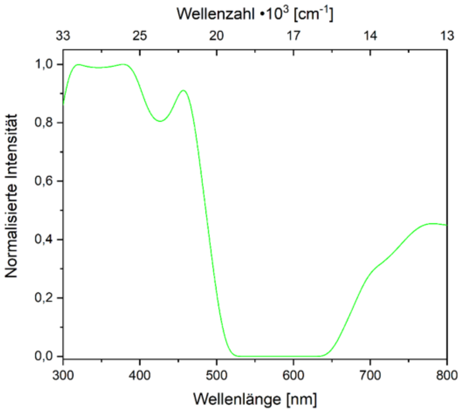

Fig. S3 | UV/vis spectrum of 3b in 1,2-$C_6H_4F_2$.

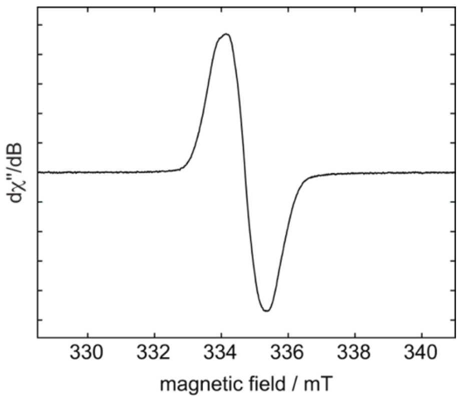

Fig. S4 | CW X-Band (9.85 GHz) EPR-spectrum of 3b at room temperature (*g* = 2.0023).

Synthesis and characterization of 4a

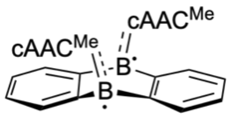

A suspension of **2a** (1.00 g, 1.10 mmol) in toluene (10 mL) was cooled to –78 °C and reacted with $[Mg(thf)_3][C_{14}H_{10}]$ (456 mg, 1.21 mmol). After 1 h, the mixture was allowed to warm to room temperature, and stirred for 2 h. All volatiles were removed *in vacuo*, and the residue was extracted into benzene (3 x 10 mL). All volatiles of the filtrate were removed *in vacuo*, and anthracene removed by sublimation (10^{-6} mbar, 70 °C, 16 h). Crystals suitable for X-ray diffraction were obtained by slow evaporation of solutions of **4a** in benzene into anthracene. Yield: 532 mg (715 μmol, 65%) of an orange solid that proved NMR silent.

UV/vis (benzene): λ_{max} = 311, <u>356</u>, 425.

FTMS/pESI calculated for $C_{52}H_{70}B_2N_2$: m/z = 744.57; found: m/z = 744.57.

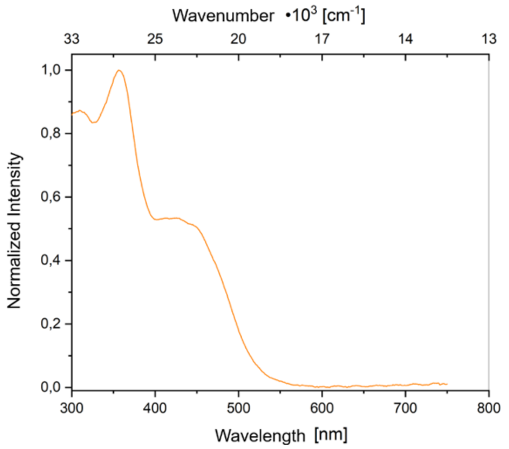

Fig. S5 | UV/vis spectrum of 4a in benzene.

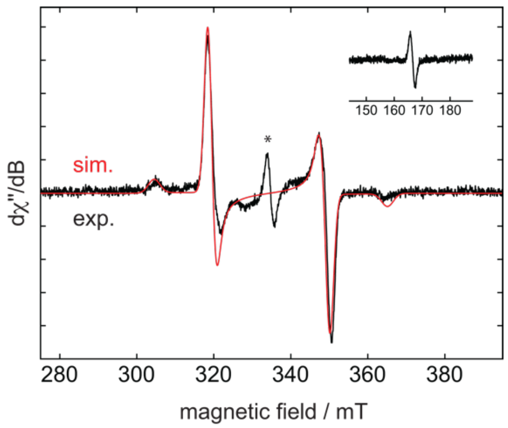

Fig. S6 | Experimental and simulated EPR spectra of diradical 4a in frozen toluene solution at 20 K. The inset shows the forbidden Δm_S = 2 half-field transition. The small center peak marked with an asterisk (*) is due to a monoradical impurity. Key parameters for the simulation of the triplet state: g_1 = 2.005, g_2 = 2.003, g_3 = 2.002, D = 0.0284 cm^{-1}, E = 0.0003 cm^{-1}.

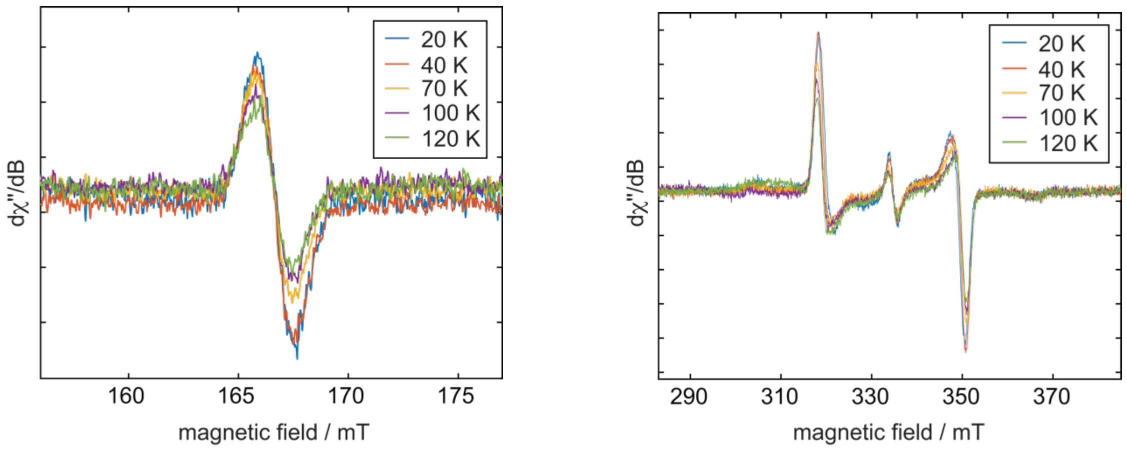

Fig. S7 | Temperature dependence of the CW X-Band EPR spectra of 4a in frozen toluene. Half-field transition (left) and the g = 2 signal (right).

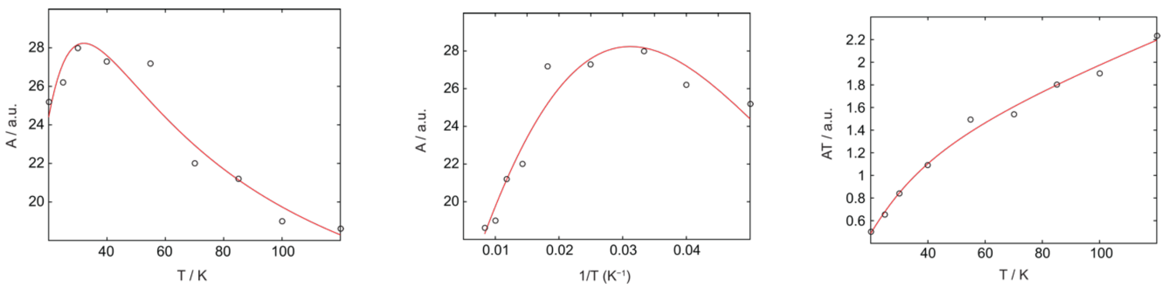

Fig. S8 | Three different representations of the temperature dependence of the double integral EPR intensity (*A*) of 4a in frozen toluene solution. Circles (○) represent the experimental results and the red line corresponds to the fit with the Bleaney-Bowers equation. Analysis of the variable temperature EPR data gives a singlet-triplet gap of Δ*E*(T-S) = 0.43 kJ/mol.

Synthesis and characterization of 4b

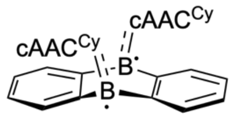

A suspension of **2b** (1.00 g, 1.02 mmol) in toluene (10 mL) was cooled to –78 °C and reacted with [Mg(thf)$_3$][$C_{14}H_{10}$] (412 mg, 1.10 mmol). After 1 h, the mixture was allowed to warm to room temperature, and stirred for 2 h. All volatiles were removed *in vacuo*, and the residue was extracted into benzene (3 x 10 mL). All volatiles of the filtrate were removed *in vacuo*, and anthracene removed by sublimation (10^{-6} mbar, 70 °C, 16 h). Crystals suitable for X-ray diffraction were obtained by slow evaporation of solutions of **4b** in benzene into anthracene. Yield: 614 mg (744 μmol, 73%) of an orange brown solid that proved NMR silent.

UV/vis (benzene): λ_{max} = <u>298</u>, 362, 430.

HRMS/LIFDI calculated for $C_{58}H_{78}B_2N_2$: m/z = 824.64; found: m/z = 824.63.

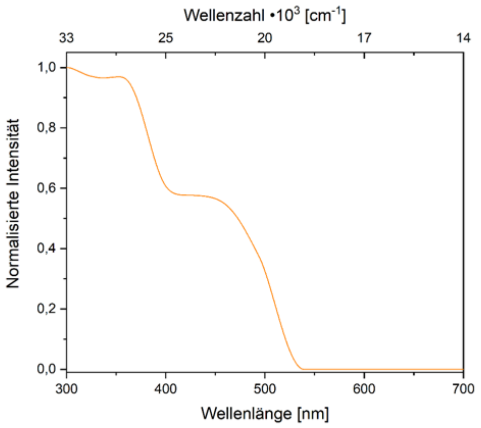

Fig. S9 | UV/vis spectrum of 4b in benzene.

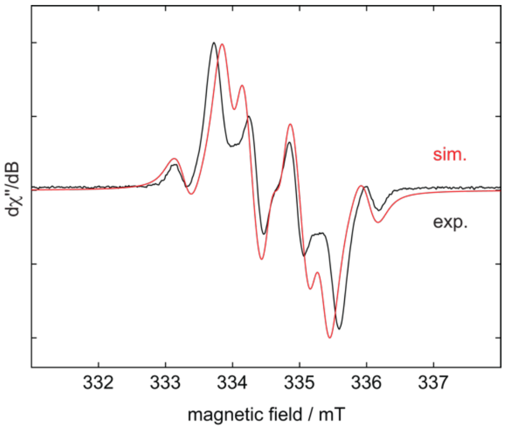

Fig. S10 | Experimental and simulated EPR spectra of diradical 4b in toluene solution. Key parameters for the simulation of the triplet state: g_{iso} = 2.003; a(N) = 19 MHz, with an exchange interaction of J = 28 MHz (coupling to boron not considered).

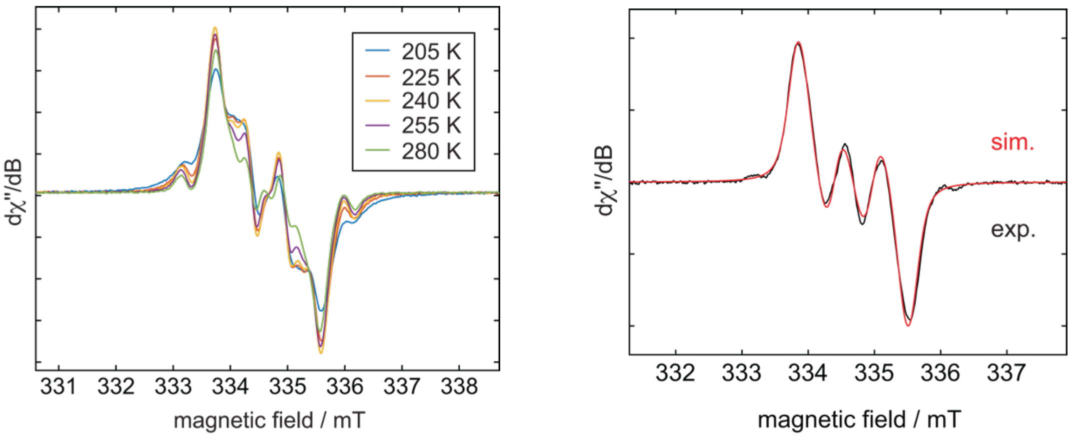

Fig. S11 | Temperature dependence of the CW X-Band EPR spectra of 4b in toluene (left). Experimental and simulated EPR spectrum of **4b** after decomposition in solution (right), most likely into a monoradical boryl species. Simulation parameters are: g_{iso} = 2.0024, a(B) = 3.9 and a(N) = 16 MHz.

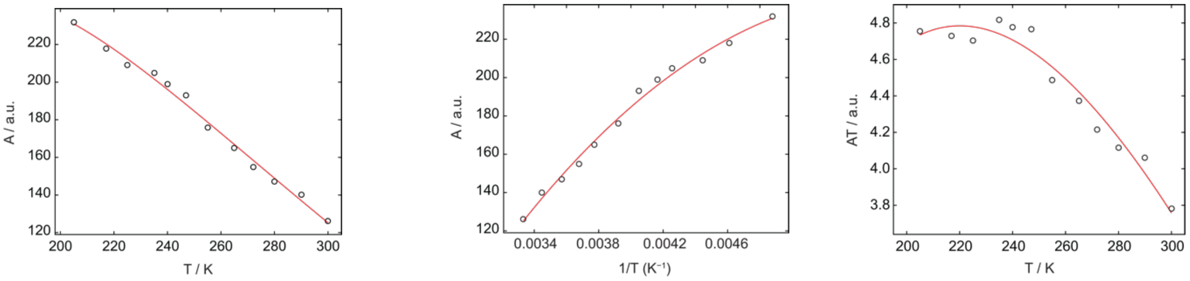

Fig. S12 | Three different representations of the temperature dependence of the double integral EPR intensity (*A*) of 4b in frozen toluene solution. Circles (○) represent the experimental results and the red line corresponds to the fit with the Bleaney-Bowers equation. Analysis of the variable temperature EPR data gives a singlet-triplet gap of Δ*E*(T-S) = 2.3 kJ/mol.

Attempted synthesis of 5a

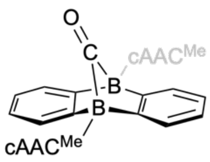

A solution of **4a** (30.0 mg, 40.3 *μ*mol) in benzene (0.5 mL) was degassed by three freeze-pump-thaw cycles, and one atmosphere of CO gas was introduced. The mixture was stirred for two days at room temperature, after which time all volatiles were removed *in vacuo*. The residue was extracted into hexanes, filtered and all volatiles were again removed under reduced pressure to afford a red solid. ^{11}B NMR spectroscopy indicated the generation of **5a**, however, we were not able to isolate this species analytically pure.

^{11}B NMR (128.4 MHz, C_6D_6): *δ* = –0.4.

Synthesis and characterization of 5b

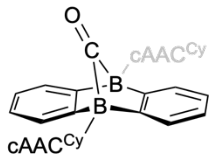

A solution of **4b** (30.0 mg, 36.3 *µ*mol) in benzene (0.5 mL) was degassed by three freeze-pump-thaw cycles, and one atmosphere of CO gas was introduced. The mixture was stirred for two days at room temperature, after which time all volatiles were removed *in vacuo*. The residue was extracted into hexanes, filtered and all volatiles were again removed under reduced pressure. Single crystals suitable for X-ray diffraction were obtained by slow evaporation of saturated benzene solutions of **5b**. Yield: 19.5 mg (22.9 *µ*mol, 63%) of a red solid.

^{1}H NMR (400.1 MHz, C_6D_6): δ = 7.68 (dd, 2H, $^{3}J_{HH}$ = 8.4 Hz, $^{4}J_{HH}$ = 1.4 Hz, $C_{anthracene}$-*H*), 7.23 (t, 2H, $^{3}J_{HH}$ = 15.2 Hz, $^{4}J_{HH}$ = 7.9 Hz, Ar_{para}-*H*), 7.12 (m, $C_{anthracene}$-*H*), 7.03 (d, 4H, $^{3}J_{HH}$ = 7.6 Hz, Ar_{meta}-*H*), 6.31 (dd, $^{3}J_{HH}$ = 8.9 Hz, $^{4}J_{HH}$ = 2.3 Hz, $C_{anthracene}$-*H*), 4.99 (dd, 2H, $^{3}J_{HH}$ = 10.1 Hz, $^{4}J_{HH}$ = 1.5 Hz, $C_{anthracene}$-*H*), 3.42 (m, C_{iPr}-*H*), 3.38 (m, C_{iPr}-*H*), 3.17 (m, 3H, $C_{Cy}H_2$), 2.99 (m, 3H, $C_{Cy}H_2$), 2.02 (m, 6H, $C_{Cy}H_2$), 1.84 (d, 4H, $^{3}J_{HH}$ = 13.2 Hz, CH_2), 1.64 (m, $C_{Cy}H_2$), 1.26 (s, 12H, $C(CH_3)_2$), 1.18 (d, 12H, $^{3}J_{HH}$ = 6.9 Hz, $C_{iPr}(CH_3)_2$), 1.14 (d, 12H, $^{3}J_{HH}$ = 6.7 Hz, $C_{iPr}(CH_3)_2$).

^{13}C NMR (100.6 MHz, C_6D_6): δ = 148.2 ($C_{i\text{-aryl}}$), 147.4 ($C_{i\text{-aryl}}$), 136.8 ($C_{q\text{-aryl}}$), 129.7 ($C_{p\text{-aryl}}$), 129.4 ($C_{anthracene}$), 128.6 ($C_{anthracene}$), 125.4 ($C_{m\text{-aryl}}$), 121.1 ($C_{anthracene}$), 120.6 ($C_{anthracene}$), 76.8 ($C(CH_3)_2$), 45.5 (CH_2), 38.3 ($C_{Cy}H_2$), 33.3 ($C_{Cy}H_2$), 29.5 ($C_{iPr}(CH_3)_2$), 28.8 ($C_{iPr}(CH_3)_2$), 24.6 ($C_{iPr}(CH_3)_2$), 23.6 ($C_{iPr}(CH_3)_2$).

^{11}B NMR (128.4 MHz, C_6D_6): δ = –2.2.

UV/vis (benzene): λ_{max} = 283, <u>463</u>.

IR (solid): ν(CO) = 1690 cm^{-1}.

HRMS/LIFDI calculated for $C_{59}H_{78}B_2N_2O$: m/z = 852.63; found: m/z = 852.63, 824.63 (**7b**).

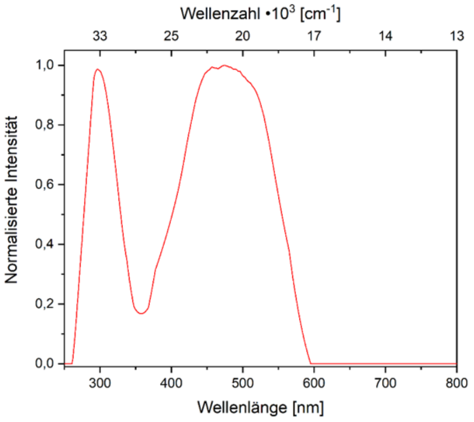

Fig. S13 | UV/vis spectrum of 5b in benzene.

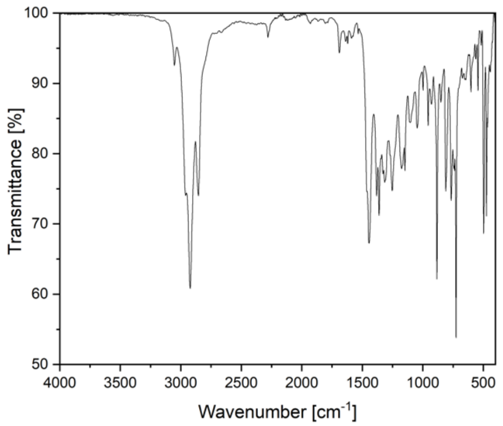

Fig. S14 | IR spectrum of 5b in the solid state.

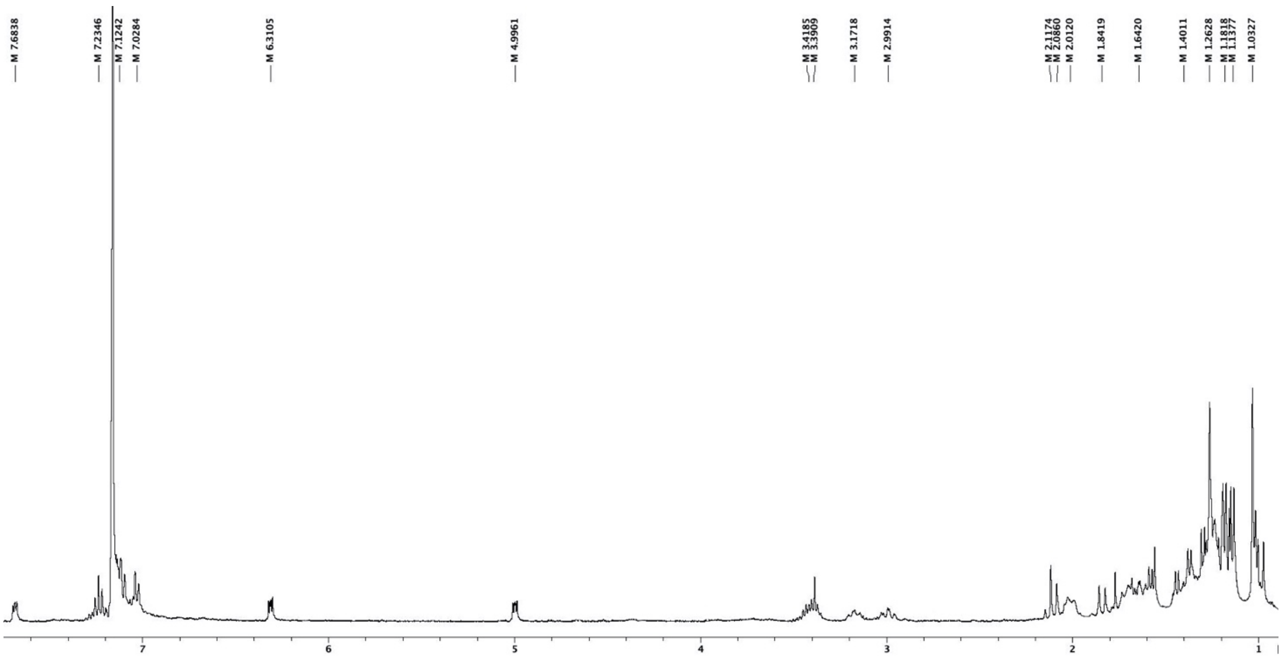

Fig. S15 | ^{1}H NMR spectrum of 5b in C_6D_6.

S2 X-ray diffraction data

General remarks: The crystal data of **3b** and **4a** were collected on a BRUKER D8 QUEST diffractometer with a CMOS area detector and multi-layer mirror monochromated $Mo_{K\alpha}$ radiation. The crystal data of **4b** was collected on a XTRALAB Synergy Dualflex diffractometer with a Hybrid Pixel Array detector and multi-layer mirror monochromated $Cu_{K\alpha}$ radiation. The crystal data of **5b** was collected on a BRUKER SMART APEX 1 diffractometer with a CCD area detector and graphite monochromated $Mo_{K\alpha}$ radiation. The structures were solved using intrinsic phasing method (SHELXT),[5] refined with the SHELXL program,[6] and expanded using Fourier techniques. All non-hydrogen atoms were refined anisotropically. Hydrogen atoms were included in structure factors calculations. All hydrogen atoms were assigned to idealized geometric positions.

Crystal data for **3b**: $C_{68}H_{90}B_2Br_3F_2MgN_2O$, M_r = 1275.07, green block, 0.589×0.366×0.296 mm^3, Monoclinic space group *Pc*, a = 16.361(4) Å, b = 10.871(3) Å, c = 18.518(5) Å, β = 109.717(12)°, V = 3100.3(13) Å^3, Z = 2, ρ_{calcd} = 1.366 g·cm^{-3}, μ = 2.012 mm^{-1}, F(000) = 1330, T = 100(2) K, R_1 = 0.0316, wR^2 = 0.0560, 12065 independent reflections [2θ≤52.042°] and 796 parameters. CCDC 2006615.

Crystal data for **4a**: $C_{111}H_{148}B_4N_4$, M_r = 1581.57, orange block, 0.202×0.122×0.113 mm^3, Triclinic space group $P\bar{1}$, a = 13.546(7) Å, b = 17.086(10) Å, c = 23.711(14) Å, α = 69.589(12)°, β = 77.133(11)°, γ = 67.339(15)°, V = 4721(5) Å^3, Z = 2, ρ_{calcd} = 1.113 g·cm^{-3}, μ = 0.062 mm^{-1}, F(000) = 1724, T = 100(2) K, R_1 = 0.2200, wR^2 = 0.2586, 16588 independent reflections [2θ≤50.046°] and 1106 parameters. CCDC 2006616.

Crystal data for **4b**: $C_{58}H_{78}B_2N_2$, M_r = 824.84, orange plate, 0.167× 0.112× 0.032 mm^3, Triclinic space group $P\bar{1}$, a = 9.7542(2) Å, b = 12.6657(2) Å, c = 22.2196(4) Å, α = 100.352(2)°, β = 97.137(2)°, γ = 99.894(2)°, V = 2625.95(9) Å^3, Z = 2, ρ_{calcd} = 1.043 g·cm^{-3}, μ = 0.434 mm^{-1}, F(000) = 900, T = 100(2) K, R_1 = 0.0628, wR^2 = 0.1479, 11044 independent reflections [2θ≤155,102°] and 603 parameters. CCDC 2006614.

Crystal data for **5b**: $C_{84}H_{101}B_2N_2O$, M_r = 1176.28, violet block, 0.365×0.294×0.274 mm^3, Monoclinic space group $P2_1/n$, a = 12.509(9) Å, b = 35.90(2) Å, c = 16.214(8) Å, β = 111.65(2)°, V = 6767(7) Å^3, Z = 4, ρ_{calcd} = 1.155 g·cm^{-3}, μ = 0.066 mm^{-1}, F(000) = 2548, T = 100(2) K, R_1 = 0.0848, wR^2 = 0.1565, 13329 independent reflections [2θ≤52.042°] and 1102 parameters. CCDC 2006613.

Crystallographic data have been deposited with the Cambridge Crystallographic Data Center as supplementary publication no. CCDC 2006613-2006616. These data can be obtained free of charge from The Cambridge Crystallographic Data Centre *via* www.ccdc.cam.ac.uk/data_request/cif.

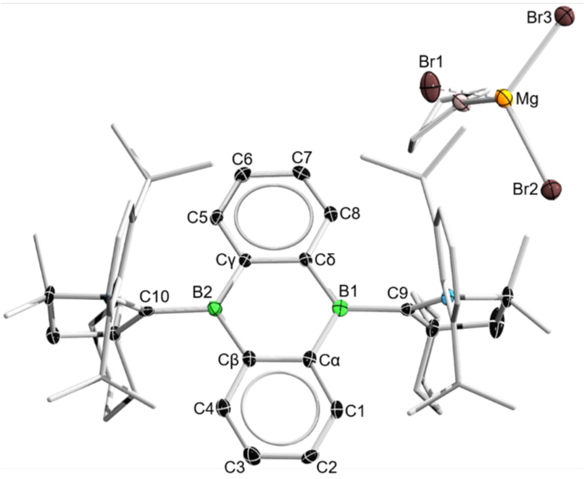

Fig. S16 | Molecular structure of 3b in the solid state. Thermal displacement parameters are displayed at the 50%-probability level. Hydrogen atoms, co-crystallized solvent molecules, and some of the thermal ellipsoids of the cAAC^{Cy} ligands are omitted for clarity. Selected bond lengths (Å) and angles (°): B1—Cα: 1.538(5), B1—Cδ: 1.539(5), B1—C9: 1.638(5), B2—Cβ: 1.542(5), B2—C10: 1.632(5), B2—Cγ: 1.548(5), Cα—Cβ: 1.446(4), Cγ—Cδ: 1.444(4), Cα —C1: 1.416(4), C1 —C2: 1.370(4), C2 —C3: 1.400(5), C3 —C4: 1.377(4), C4 —Cβ: 1.409(4), Cγ—C5: 1.410(4), C5 —C6: 1.372(4), C6 —C7: 1.402(4), C7 —C8: 1.370(4), C8–Cδ: 1.419(4), Br1—Mg: 2.4822(1), Br2—Mg: 2.4484(1), Br3—Mg: 2.4601(1), Cα—B1—C9: 120.8(3), Cα —B1—Cδ: 118.8(3), B1—Cδ—Cγ: 120.1(3), Cγ—B2—Cβ: 118.6(3), Cβ—B2—C10: 120.9(3), B2—Cβ—Cα: 120.6(3), B1—Cα —Cβ: 120.4(3), Cα—C1—C2: 123.3(3), C1—C2—C3: 119.6(3), C3—C4—Cβ: 123.2(3), Cγ—C5—C6: 123.9(3), C5—C6—C7: 119.0(3), C6—C7—C8: 119.0(3), C7—C8—Cδ: 123.4(3), Br1—Mg—Br2: 117.54(5), Br1—Mg—Br3: 112.04(5), Br —Mg —O: 103.67(8).

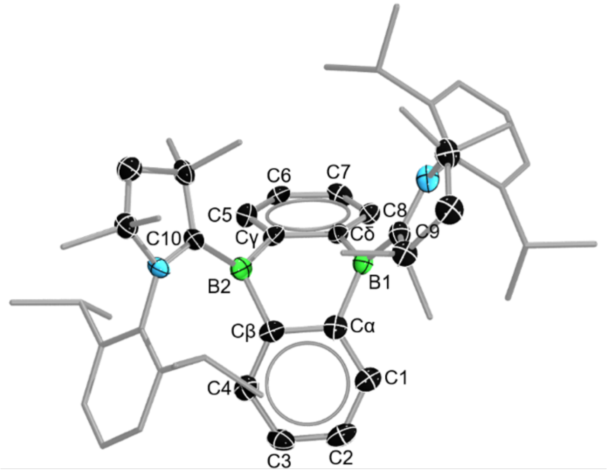

Fig. S17 | Molecular structure of 4a in the solid state. Thermal displacement parameters are displayed at the 50%-probability level. The asymmetric unit contains two independent molecules, only one is displayed. Hydrogen atoms, co-crystallized solvent molecules, and some of the thermal ellipsoids of the cAAC^{Me} ligands are omitted for clarity. Selected bond lengths (Å) and angles (°): B1—C9: 1.527(6), B1—Cα: 1.583(7), B1—Cδ: 1.585(7), B2—C10: 1.52(1), B2—Cβ: 1.569(8), B2—Cγ: 1.587(9), Cα—Cβ: 1.441(7), Cγ—Cδ: 1.437(9), Cα—C1:1.394(7), C1—C2: 1.395(6), C2—C3: 1.395(6), C3—C4: 1.390(8), C4—Cβ: 1.400(5), Cγ—C5: 1.393(7), C5—C6: 1.383(7), C6—C7: 1.376(9), C7—C8: 1.406(7), C8—Cδ: 1.400(7), C9—B1—Cα: 123.3(5), Cα—B1—Cδ: 108.1(5), Cβ—B2—C10: 128.2(5), Cβ—B2—Cγ: 108.7(5), B1—Cα—Cβ: 116.2(4), Cα—Cβ—B2: 112.5(4), B2—Cγ—Cδ: 115.8(5), Cγ—Cδ—B1: 112.8(4), B1—Cα—C1: 112.8(4), Cα—C1—C2: 122.1(4), C1—C2—C3: 119.4(5), C2—C3—C4: 120.2(5), C3—C4—Cβ: 121.7(5), B2—Cγ—C5: 125.7(5), Cγ—C5—C6: 122.9(5), C5—C6—C7: 119.5(5), C6—C7—C8: 119.6(5), C7—C8—Cδ: 121.8(5).

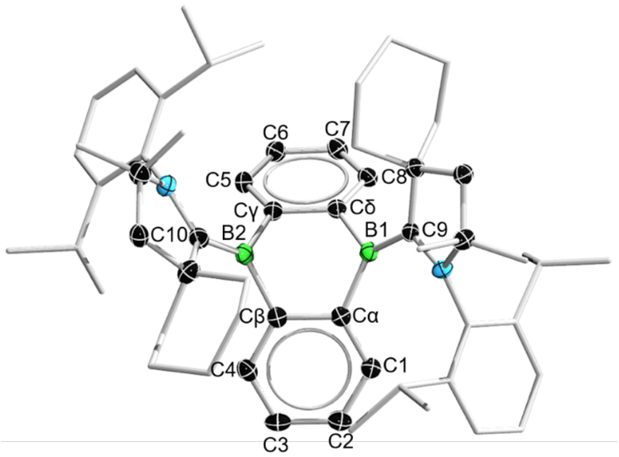

Fig. S18 | Molecular structure of 4b in the solid state. Thermal displacement parameters are displayed at the 50%-probability level. Hydrogen atoms, co-crystallized solvent molecules, and some of the thermal ellipsoids of the cAAC^{Cy} ligands are omitted for clarity. Selected bond lengths (Å) and angles (°): B1—C9: 1.531(2), B1—Cα: 1.589(3), B1—Cδ: 1.595(2), B2—C10: 1.528(2), B2—Cβ: 1.593(2), B2—Cγ: 1.588(3), Cα—Cβ: 1.429(2), Cγ—Cδ: 1.431(2), Cα—C1: 1.400(2), C1—C2: 1.391(3), C2—C3: 1.391(3), C3—C4: 1.394(2), C4—Cβ: 1.401(2), Cγ—C5: 1.399(2), C5—C6: 1.394(3), C6—C7: 1.384(2), C7—C8: 1.393(2), C8—Cδ: 1.401(3), C9—B1—Cα: 127.4(1), Cα—B1—Cδ: 107.2(1), Cβ—B2—C10: 125.1(1), Cβ—B2—Cγ: 106.8(1), B1—Cα—Cβ: 113.5(1), Cα—Cβ—B2: , B2—Cγ—Cδ: 117.0(1), Cγ—Cδ—B1: 117.0(1), B1—Cα—C1: 127.5(1), Cα—C1—C2: 121.6(1), C1—C2—C3: 120.0(2), C2—C3—C4: 119.7(1), C3—C4—Cβ: 121.6(1), B2—Cγ—C5: 127.5(1), Cγ—C5—C6: 121.4(1), C5—C6—C7: 120.0(2), C6—C7—C8: 119.7(2), C7—C8—Cδ: 121.6(2).

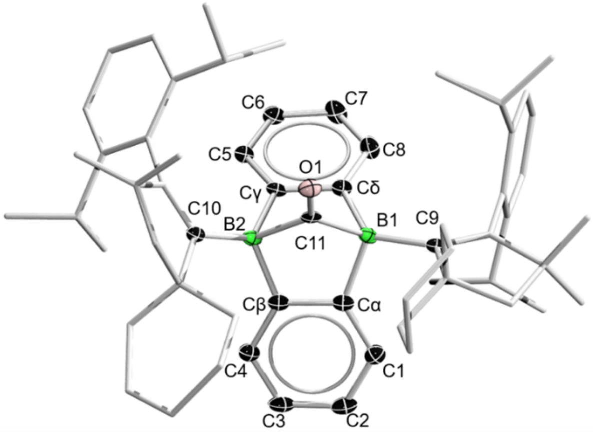

Fig. S19 | Molecular structure of 5b in the solid state. Thermal displacement parameters are displayed at the 50%-probability level. The asymmetric unit contains two independent molecules, only one is displayed. Hydrogen atoms, co-crystallized solvent molecules, and some of the thermal ellipsoids of the cAAC^{Cy} ligands are omitted for clarity. Selected bond lengths (Å) and angles (°): B1—C9: 1.630(3), B1—Cα: 1.637(3), B1—Cδ: 1.649(3), B2—C10: 1.589(3), B2—Cβ: 1.609(3), B2—Cγ: 1.630(3), Cα—Cβ: 1.417(3), Cγ—Cδ: 1.433(3), Cα—C1: 1.397(3), C1—C2: 1.396(3), C2—C3: 1.384(3), C3—C4: 1.393(3), C4—Cβ: 1.399(3), Cγ—C5: 1.399(3), C5—C6: 1.392(3), C6—C7: 1.377(3), C7—C8: 1.394(3), C8—Cδ: 1.399(3), B1—C11: 1.695(3), C11—O1: 1.205(2), C11—B2: 1.755(3), C9—B1—Cα: 106.8(2), Cα—B1—Cδ: 105.5(2), Cα—B1—C11: 98.9(1), Cβ—B2—C10: 121.9(2), Cβ—B2—Cγ: 104.0(2), Cβ—B2—C11: 99.3(1), B1—Cα—Cβ: 109.8(2), Cα—Cβ—B2: 110.7(2), B2—Cγ—Cδ: 110.5(2), Cγ—Cδ—B1: 109.0(2), B1—Cα—C1: 130.7(2), Cα—C1—C2: 120.9(2), C1—C2—C3: 119.7(2), C2—C3—C4: 120.1(2), C3—C4—Cβ: 121.2(2), B2—Cγ—C5: 130.2(2), Cγ—C5—C6: 121.4(2), C5—C6—C7: 121.4(2), C6—C7—C8: 121.4(2), C7—C8—Cδ: 121.4(2), B1—C11—B2: 94.9(1), B1—C11—O1: 133.1(2), B2—C11—O1: 130.6(2).

S3 Computational details

General remarks: Initially, we performed geometry optimization and hessian calculations for the closed-shell singlet (CS), open-shell singlet (OS) and triplet (T) states of **4a** and **4b** at the (U)B3LYP[7]-D3[8](BJ)[9]/def2-SVP[10] level of theory. We collected the vertical ΔE and adiabatic ΔE_0 energy gaps between the states, the former calculated at the equilibrium geometry of the OS states. The DFT calculations pointed the OS states as the ground states of both systems, with the triplet states lying less than 0.15 kcal/mol above. Additionally, we also optimized the geometry of **5b** at the B3LYP-D3(BJ)/def2-SVP level of theory. All systems were characterized as minimum energy structures by vibrational frequency calculations, which indicated that all Hessian eigenvalues were positive. In order to confirm the biradical character[11] of the singlet diboraanthracenes studied herein, as well as to validate the computed singlet-triplet gaps obtained by DFT, single-point calculations were performed for **4a** using high-level complete active space self-consistent field (CASSCF)[12] and N-electron valence state second-order perturbation theory (NEVPT2)[13] calculations. Due to the large molecular size, these calculations were done using the Resolution of the Identity (RI)[14] approximation. The CASSCF calculations were performed for two distinct active spaces: one composed of 2 electrons and 2 orbitals, CASSCF(2,2); and a second one containing 6 electrons and 6 orbitals, CASSCF(6,6). For an accurate determination of the adiabatic singlet-triplet gaps, RI-NEVPT2 calculations were performed using reference CASSCF(2,2) and CASSCF(6,6) wavefunctions for both the singlet and triplet multiplicities. All DFT calculations were performed with the Gaussian 16, Revision B.01 software.[15] CASSCF and RI-NEVPT2 calculations were performed with the Orca 4.1.1 software.[16] Pictures of molecular structures, orbitals and densities were visualized and generated with Chemcraft, CYLview,[17] and Gaussview.

The biradical character index, *y*

The y index,[18] which can vary from 0 (closed-shell system) to 1 (pure biradical state), was obtained for **4a** using the natural orbital occupancy numbers (NOON)[19] of the highest occupied (HONO) and lowest unoccupied (LUNO) natural orbitals from the CASSCF calculations, according to the following expression[18]:

$$y = 1 - \frac{2T}{1 + T^2} \qquad (S1)$$

where T is the orbital overlap of HONO and LUNO, and is given by:

$$T = \frac{NOON_{HONO} - NOON_{LUNO}}{2} \qquad (S2)$$

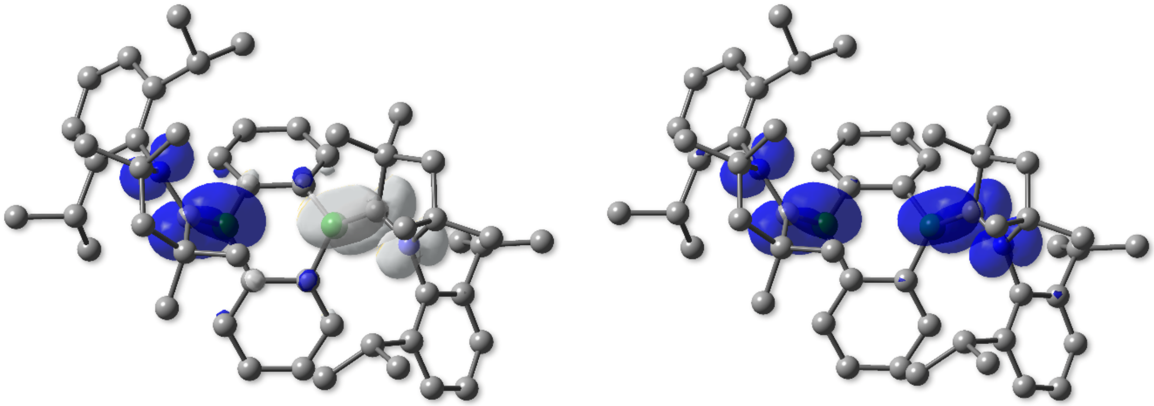

Fig. S20 | Mulliken spin density plots of 7a. Blue colors illustrate the alpha spin density, and white colors are used for the beta spin density. Level of theory: B3LYP-D3(BJ)/def2-SVP; isovalues: 0.004 au.

Table S1 | Electronic energies of open-shell singlet (OS), closed-shell singlet (CS) and triplet states of 4a and 4b. The vertical (ΔE(T-OS) and ΔE(OS-CS)) and adiabatic (ΔE_0(T-OS) and ΔE_0(OS-CS)) energy gaps, as well as the average values of $<S^2>$ for the OS state before and after (values in parentheses) annihilation of the first spin contaminant, are also shown.

	**4a**	**4b**
**Electronic energies, E, at the open-shell singlet optimized structure**		
OS, E_h	-2181.683400	-2415.024889
CS, E_h	-2181.655968	-2414.998135
TS, E_h	-2181.683221	-2415.024678
$<S^2>$ (OS)	1.00 (0.05)	1.00 (0.05)
Vertical ΔE(T-OS), kcal/mol	0.11	0.13
Vertical ΔE(OS-CS), kcal/mol	-17.21	-16.79
**ZPE-corrected energies, E+ZPE**		
OS, E_h	-2180.575091	-2413.783645
CS, E_h	-2180.555035	-2413.764114
TS, E_h	-2180.574861	-2413.783413
Adiabatic ΔE_0(T-OS), kcal/mol	0.14	0.15
Adiabatic ΔE_0(OS-CS), kcal/mol	-12.59	-12.26

Table S2 | CASSCF(2,2)/RI-NEVPT2 results of the singlet and triplet states of 4a using the def2-SVP basis set.

CASSCF(2,2)/def2-SVP	
Electronic energy (singlet), E_h	-2166.526832
Electronic energy (triplet), E_h	-2166.527022
Weight of the 2 0 configuration (singlet)	0.53858
Weight of the 0 2 configuration (singlet)	0.46142
Occupation of the HONO (ON^{HONO}, singlet)	1.0772
Occupation of the LUNO (ON^{LUNO}, singlet)	0.9228
HONO-LUNO orbital overlap (T, singlet)	0.0772
Biradical character (y, singlet)	0.847 (84.7%)
**RI-NEVPT2/CASSCF(2,2)/def2-SVP**	
Electronic energy (singlet), E_h	-2174.430054
Electronic energy (triplet), E_h	-2174.429979
Adiabatic singlet-triplet gap, ΔE_0(T-S), kcal/mol	0.05

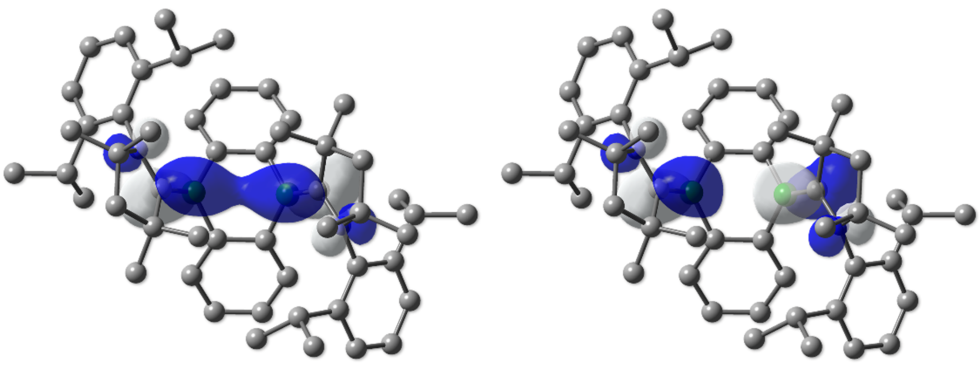

HONO
NOON: 1.077

LUNO
NOON: 0.9228

Fig. S21 | CASSCF(2,2) active space natural orbitals of 4a. Natural orbital occupation numbers (NOON) are also shown. Level of theory: CASSCF(2,2)/def2-SVP from optimized structures at UB3LYP-D3(BJ)/def2-SVP. CASSCF calculations were performed with the Resolution of the Identity (RI) approximation.

Table S3 | CASSCF(6,6)/RI-NEVPT2 results of the singlet and triplet states of 4a using the def2-SVP basis set.

**CASSCF(6,6)/def2-SVP**	
Electronic energy (singlet), E_h	-2166.538685
Electronic energy (triplet), E_h	-2166.538864
Weight of the 2 2 2 0 0 0 configuration (singlet)	0.53042
Weight of the 2 2 0 2 0 0 configuration (singlet)	0.45298
Occupation of the HONO (ON^{HONO}, singlet)	1.0789
Occupation of the LUNO (ON^{LUNO}, singlet)	0.9213
HONO-LUNO orbital overlap (T, singlet)	0.0788
Biradical character (y, singlet)	0.843 (84.3%)
**RI-NEVPT2/CASSCF(6,6)/def2-SVP**	
Electronic energy (singlet), E_h	-2174.436983
Electronic energy (triplet), E_h	-2174.436875
Adiabatic singlet-triplet gap, ΔE_0(T-S), kcal/mol	0.07

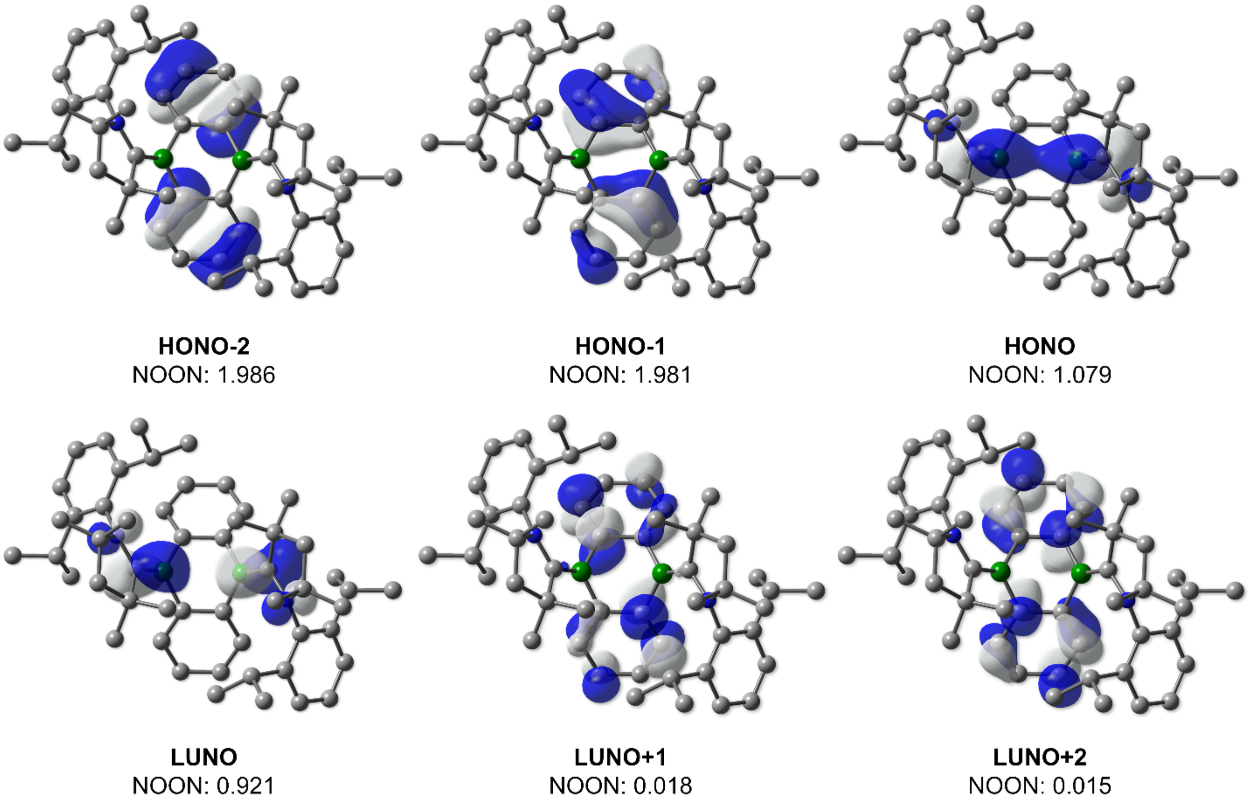

Fig. S22 | CASSCF(6,6) active space natural orbitals of 4a. Natural orbital occupation numbers (NOON) are also shown. Level of theory: CASSCF(6,6)/def2-SVP from optimized structures at UB3LYP-D3(BJ)/def2-SVP. CASSCF calculations were performed with the Resolution of the Identity (RI) approximation.

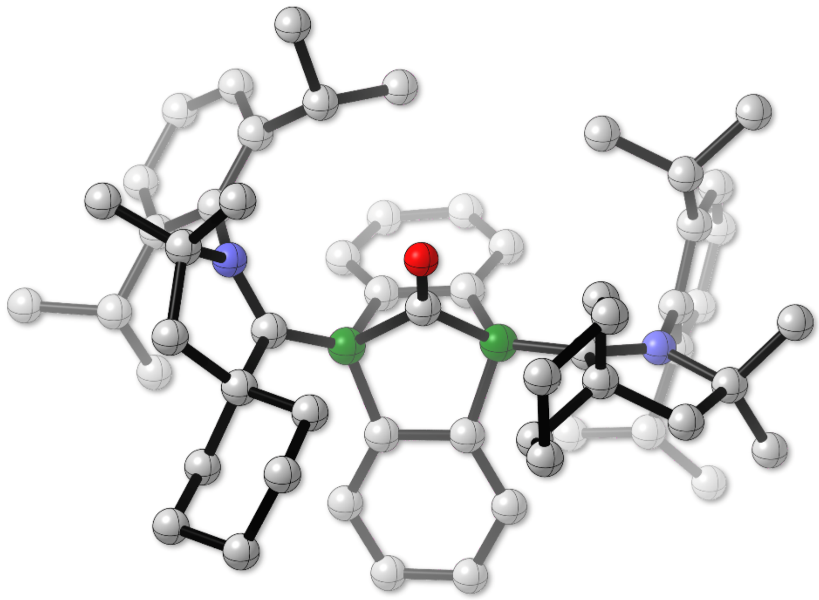

Fig. S23 | 3D image of the optimized structure of 5b. Level of theory: B3LYP-D3(BJ)/def2-SVP.

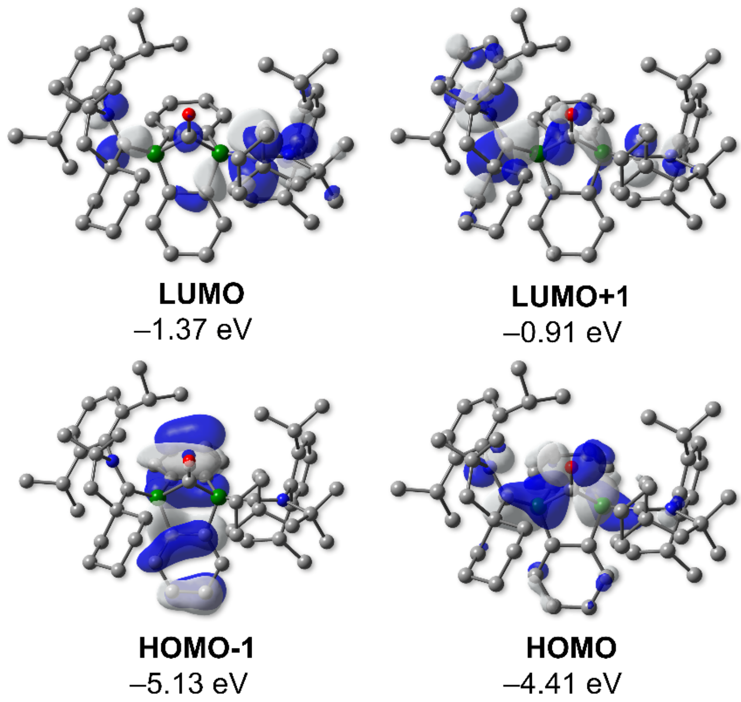

Fig. S24 | Frontier Kohn-Sham Molecular Orbitals of 5b. Level of theory: B3LYP-D3(BJ)/def2-SVP; isovalues: 0.04 au.

S4 Cartesian Coordinates

4a (closed-shell singlet), B3LYP-D3(BJ)/def2-SVP
E+ZPE = –2180.555035 E_h
Lowest frequency = 39.63 cm^{-1}

N	-2.975739000	-0.508641000	0.948824000
N	2.975674000	0.508598000	0.948769000
C	3.952114000	-0.156108000	0.127115000
C	2.333747000	-1.242797000	-2.384504000
C	1.282382000	-0.751360000	-1.602528000
C	0.059707000	-1.465225000	-1.549463000
C	-0.067127000	-2.665940000	-2.260539000
C	-3.354285000	-0.997709000	2.323793000
C	5.214785000	-2.118184000	-0.513585000
C	4.702950000	0.626284000	-0.788396000
C	-1.405971000	-2.212895000	1.402027000
C	0.066989000	2.665908000	-2.260224000
C	-1.282557000	0.751209000	-1.602635000
C	2.194289000	-2.443904000	-3.092358000
C	0.998294000	-3.159235000	-3.026288000
C	-1.822100000	-1.063761000	0.464197000
C	1.405718000	2.212620000	1.402266000
C	-0.998319000	3.159247000	-3.026100000
C	5.698747000	-0.002120000	-1.545446000
C	-3.952166000	0.156230000	0.127298000
C	4.204750000	-1.538315000	0.265040000
C	5.960765000	-1.363385000	-1.411883000
C	-4.703063000	-0.626037000	-0.788256000
C	-3.404019000	2.434984000	1.192463000
C	1.821881000	1.063570000	0.464339000
C	-0.059910000	1.465100000	-1.549316000
C	-4.204610000	1.538472000	0.265247000
C	-5.698814000	0.002492000	-1.545263000
C	-0.101294000	-1.879583000	2.142092000
C	4.291651000	-3.060255000	2.280072000
C	3.354232000	0.997461000	2.323795000
C	4.864486000	1.174061000	2.476937000
C	-2.333788000	1.242667000	-2.384784000
C	3.404468000	-2.434877000	1.192471000
C	4.454004000	2.109962000	-1.020712000
C	2.848536000	0.048703000	3.424341000
C	2.608135000	2.338815000	2.365770000
C	2.645322000	-3.522845000	0.420090000
C	1.207948000	3.554221000	0.672210000
C	-2.645211000	3.523054000	0.419888000
C	-4.454160000	-2.109702000	-1.020703000
C	-2.608454000	-2.339216000	2.365418000
C	0.100945000	1.879310000	2.142178000

C	-5.214640000	2.118460000	-0.513297000
C	-5.960734000	1.363766000	-1.411593000
C	-1.208072000	-3.554414000	0.671849000
B	-1.078311000	-0.618775000	-0.805609000
C	-4.290810000	3.060233000	2.280456000
C	-2.194255000	2.443852000	-3.092487000
C	-5.732295000	-2.953264000	-0.903639000
C	-3.774991000	-2.338286000	-2.380129000
C	5.732006000	2.953622000	-0.902921000
C	-2.848272000	-0.049345000	3.424532000
C	3.775468000	2.338699000	-2.380426000
B	1.078034000	0.618559000	-0.805427000
C	-4.864560000	-1.174035000	2.477032000
H	1.983914000	-3.085169000	-0.336627000
H	2.034617000	-4.123017000	1.112873000
H	3.336754000	-4.208085000	-0.094726000
H	2.655026000	-1.804963000	1.681636000
H	5.004896000	-3.779248000	1.846446000
H	3.677421000	-3.605180000	3.015072000
H	4.880329000	-2.302512000	2.819662000
H	5.406103000	-3.189129000	-0.422352000
H	6.741079000	-1.835072000	-2.013747000
H	6.278217000	0.588277000	-2.257744000
H	3.761605000	2.462053000	-0.248523000
H	6.448753000	2.725621000	-1.707127000
H	6.246025000	2.786851000	0.056089000
H	5.487631000	4.025323000	-0.977484000
H	4.438136000	2.036022000	-3.206959000
H	3.529864000	3.403999000	-2.518216000
H	2.846335000	1.760114000	-2.468719000
H	1.796653000	-0.217519000	3.275629000
H	2.943174000	0.546032000	4.401751000
H	3.433863000	-0.876240000	3.464540000
H	5.272142000	1.863723000	1.727587000
H	5.396969000	0.217000000	2.385787000
H	5.074293000	1.589557000	3.474281000
H	2.299896000	2.610731000	3.385611000
H	3.281491000	3.132460000	2.006915000
H	-0.679319000	1.622811000	1.417682000
H	-0.236434000	2.740291000	2.741948000
H	0.227650000	1.024424000	2.816522000
H	0.295641000	3.558368000	0.069370000
H	2.056587000	3.786605000	0.012361000
H	1.130391000	4.360179000	1.421119000
H	1.003931000	3.226118000	-2.234325000
H	-0.886948000	4.097273000	-3.577102000
H	-3.026932000	2.819053000	-3.693617000
H	-3.276574000	0.702033000	-2.455160000
H	-2.034314000	4.123229000	1.112499000
H	-3.336874000	4.208274000	-0.094648000
H	-1.984031000	3.085476000	-0.337084000

H	-3.676320000	3.605160000	3.015237000
H	-4.879215000	2.302416000	2.820244000
H	-5.004279000	3.779204000	1.847163000
H	-2.654359000	1.805070000	1.681288000
H	-5.405814000	3.189431000	-0.422055000
H	-6.741014000	1.835556000	-2.013420000
H	-6.278335000	-0.587815000	-2.257594000
H	-2.845737000	-1.759804000	-2.467865000
H	-4.437217000	-2.035385000	-3.206934000
H	-3.529453000	-3.403594000	-2.517985000
H	-6.246741000	-2.786620000	0.055163000
H	-5.487986000	-4.024975000	-0.978282000
H	-6.448653000	-2.725050000	-1.708132000
H	-3.762140000	-2.461975000	-0.248257000
H	-3.433418000	0.875695000	3.465077000
H	-1.796351000	0.216709000	3.275762000
H	-2.942880000	-0.546935000	4.401812000
H	-5.396853000	-0.216843000	2.386162000
H	-5.074365000	-1.589731000	3.474294000
H	-5.272425000	-1.863432000	1.727552000
H	-3.281919000	-3.132660000	2.006318000
H	-2.300296000	-2.611447000	3.385199000
H	-2.056643000	-3.786776000	0.011904000
H	-1.130537000	-4.360446000	1.420680000
H	-0.295710000	-3.558451000	0.069090000
H	-0.228156000	-1.024829000	2.816574000
H	0.678976000	-1.622850000	1.417692000
H	0.236152000	-2.740633000	2.741725000
H	3.276580000	-0.702204000	-2.454617000
H	3.027063000	-2.819093000	-3.693360000
H	0.886977000	-4.097195000	-3.577413000
H	-1.004102000	-3.226111000	-2.234869000

4a (open-shell singlet), UB3LYP-D3(BJ)/def2-SVP
E+ZPE = –2180.575091 E_h
Lowest frequency = 35.33 cm^{-1}

N	-3.113116000	-0.521723000	0.913084000
N	3.113095000	0.521729000	0.913335000
C	4.057776000	-0.203467000	0.113876000
C	2.312119000	-1.309276000	-2.280773000
C	1.263705000	-0.761497000	-1.527101000
C	0.016848000	-1.454867000	-1.474209000
C	-0.110401000	-2.673500000	-2.160472000
C	-3.476091000	-1.007328000	2.288705000
C	5.193261000	-2.245853000	-0.514785000
C	4.838029000	0.519871000	-0.825376000
C	-1.576107000	-2.274736000	1.333016000
C	0.110652000	2.673625000	-2.160360000
C	-1.263533000	0.761603000	-1.527200000

C	2.159280000	-2.516314000	-2.970423000
C	0.947443000	-3.204584000	-2.906771000
C	-1.996953000	-1.137361000	0.382682000
C	1.576085000	2.274756000	1.333217000
C	-0.947099000	3.204727000	-2.906777000
C	5.779914000	-0.177060000	-1.591623000
C	-4.057767000	0.203468000	0.113581000
C	4.228709000	-1.597652000	0.268001000
C	5.964628000	-1.549333000	-1.438799000
C	-4.837900000	-0.519887000	-0.825759000
C	-3.394170000	2.433121000	1.223925000
C	1.996968000	1.137396000	0.382889000
C	-0.016680000	1.454974000	-1.474146000
C	-4.228840000	1.597634000	0.267772000
C	-5.779770000	0.177005000	-1.592058000
C	-0.259336000	-1.918521000	2.049077000
C	4.263972000	-3.041973000	2.335578000
C	3.475940000	1.007205000	2.289025000
C	4.988384000	1.142374000	2.467125000
C	-2.311857000	1.309411000	-2.280978000
C	3.393780000	-2.433112000	1.223950000
C	4.672535000	2.014588000	-1.066932000
C	2.923280000	0.083777000	3.390999000
C	2.769071000	2.370223000	2.310252000
C	2.604068000	-3.534548000	0.502442000
C	1.390875000	3.639583000	0.647116000
C	-2.604347000	3.534609000	0.502625000
C	-4.672311000	-2.014592000	-1.067326000
C	-2.769218000	-2.370339000	2.309880000
C	0.259190000	1.918595000	2.049074000
C	-5.193351000	2.245798000	-0.515095000
C	-5.964576000	1.549264000	-1.439214000
C	-1.390716000	-3.639524000	0.646888000
B	-1.203401000	-0.663385000	-0.833023000
C	-4.264661000	3.041946000	2.335340000
C	-2.158929000	2.516459000	-2.970592000
C	-6.002178000	-2.777793000	-0.980364000
C	-3.978891000	-2.277412000	-2.413473000
C	6.002460000	2.777693000	-0.980020000
C	-2.923529000	-0.084033000	3.390839000
C	3.979070000	2.277460000	-2.413046000
B	1.203498000	0.663458000	-0.832875000
C	-4.988550000	-1.142528000	2.466654000
H	1.942383000	-3.116339000	-0.263994000
H	1.987662000	-4.090908000	1.226088000
H	3.273496000	-4.257712000	0.010918000
H	2.660265000	-1.762068000	1.683717000
H	4.946661000	-3.804684000	1.927931000
H	3.635737000	-3.531515000	3.097264000
H	4.885365000	-2.285005000	2.837572000
H	5.325516000	-3.324434000	-0.409418000

H	6.705466000	-2.075202000	-2.045755000
H	6.380120000	0.366666000	-2.323803000
H	4.017858000	2.412022000	-0.283241000
H	6.689552000	2.498950000	-1.794199000
H	6.520854000	2.584734000	-0.028176000
H	5.824986000	3.862217000	-1.059024000
H	4.600334000	1.928144000	-3.253328000
H	3.796376000	3.355239000	-2.553063000
H	3.012567000	1.758150000	-2.473375000
H	1.873407000	-0.176717000	3.213016000
H	2.990034000	0.597861000	4.362077000
H	3.497371000	-0.845336000	3.468848000
H	5.425939000	1.818223000	1.721175000
H	5.494440000	0.170029000	2.384909000
H	5.197589000	1.553770000	3.466355000
H	2.458528000	2.663409000	3.323659000
H	3.469949000	3.139053000	1.949254000
H	-0.530795000	1.729419000	1.311725000
H	-0.065409000	2.745679000	2.701508000
H	0.361295000	1.018645000	2.667218000
H	0.471169000	3.679078000	0.055586000
H	2.238496000	3.878137000	-0.013073000
H	1.331297000	4.424889000	1.419393000
H	1.064505000	3.204800000	-2.147661000
H	-0.816546000	4.145739000	-3.447985000
H	-2.990186000	2.913396000	-3.559125000
H	-3.261940000	0.783910000	-2.359805000
H	-1.988181000	4.091020000	1.226437000
H	-3.273696000	4.257719000	0.010912000
H	-1.942422000	3.116445000	-0.263623000
H	-3.636631000	3.531446000	3.097221000
H	-4.886210000	2.284970000	2.837127000
H	-4.947224000	3.804686000	1.927535000
H	-2.660734000	1.762111000	1.683861000
H	-5.325714000	3.324362000	-0.409676000
H	-6.705402000	2.075104000	-2.046211000
H	-6.379882000	-0.366736000	-2.324303000
H	-3.012419000	-1.758052000	-2.473842000
H	-4.600212000	-1.928120000	-3.253722000
H	-3.796147000	-3.355180000	-2.553509000
H	-6.520546000	-2.584876000	-0.028496000
H	-5.824632000	-3.862303000	-1.059384000
H	-6.689324000	-2.499094000	-1.794512000
H	-4.017577000	-2.411986000	-0.283661000
H	-3.497664000	0.845039000	3.468790000
H	-1.873657000	0.176527000	3.212947000
H	-2.990313000	-0.598257000	4.361841000
H	-5.494605000	-0.170176000	2.384520000
H	-5.197837000	-1.554055000	3.465813000
H	-5.426035000	-1.818285000	1.720580000
H	-3.470036000	-3.139145000	1.948717000

H	-2.458797000	-2.663616000	3.323298000
H	-2.238219000	-3.878097000	-0.013445000
H	-1.331220000	-4.424857000	1.419144000
H	-0.470913000	-3.678946000	0.055506000
H	-0.361589000	-1.018609000	2.667253000
H	0.530744000	-1.729259000	1.311852000
H	0.065225000	-2.745615000	2.701519000
H	3.262209000	-0.783769000	-2.359477000
H	2.990607000	-2.913233000	-3.558870000
H	0.816954000	-4.145583000	-3.448018000
H	-1.064249000	-3.204684000	-2.147890000

4a (triplet), UB3LYP-D3(BJ)/def2-SVP

E+ZPE = –2180.574862 E_h

Lowest frequency = 35.21 cm^{-1}

N	-3.119165000	-0.522911000	0.910914000
N	3.119097000	0.522963000	0.911404000
C	4.062193000	-0.205156000	0.113038000
C	2.311521000	-1.313131000	-2.274500000
C	1.262850000	-0.762211000	-1.522931000
C	0.014765000	-1.454481000	-1.470538000
C	-0.112289000	-2.674353000	-2.155188000
C	-3.482189000	-1.008990000	2.286229000
C	5.192916000	-2.250796000	-0.513605000
C	4.843181000	0.515251000	-0.827895000
C	-1.583453000	-2.277395000	1.329500000
C	0.112792000	2.674550000	-2.154946000
C	-1.262579000	0.762377000	-1.523264000
C	2.158179000	-2.520483000	-2.962955000
C	0.945436000	-3.207427000	-2.899753000
C	-2.004672000	-1.140515000	0.378613000
C	1.583257000	2.277283000	1.330174000
C	-0.944662000	3.207659000	-2.899869000
C	5.782901000	-0.184758000	-1.594002000
C	-4.062287000	0.205195000	0.112559000
C	4.230247000	-1.599540000	0.268983000
C	5.964896000	-1.557199000	-1.439313000
C	-4.843125000	-0.515267000	-0.828449000
C	-3.394836000	2.432199000	1.226405000
C	2.004598000	1.140573000	0.379141000
C	-0.014511000	1.454650000	-1.470394000
C	-4.230521000	1.599562000	0.268541000
C	-5.782856000	0.184655000	-1.594622000
C	-0.266760000	-1.919661000	2.045298000
C	4.263940000	-3.039448000	2.339558000
C	3.482045000	1.008834000	2.286806000
C	4.994629000	1.142957000	2.464844000
C	-2.310967000	1.313326000	-2.275207000
C	3.394253000	-2.432097000	1.226647000

C	4.680914000	2.010114000	-1.071033000
C	2.928697000	0.086798000	3.389721000
C	2.776308000	2.372390000	2.307081000
C	2.603425000	-3.534601000	0.507947000
C	1.397309000	3.643074000	0.646265000
C	-2.603341000	3.534293000	0.507811000
C	-4.680640000	-2.010101000	-1.071609000
C	-2.776581000	-2.372610000	2.306292000
C	0.266533000	1.919372000	2.045826000
C	-5.193169000	2.250733000	-0.514145000
C	-5.964991000	1.557077000	-1.439939000
C	-1.397526000	-3.643087000	0.645394000
B	-1.207927000	-0.664497000	-0.834157000
C	-4.265009000	3.040148000	2.338611000
C	-2.157377000	2.520711000	-2.963549000
C	-6.012323000	-2.770329000	-0.986143000
C	-3.986949000	-2.273188000	-2.417578000
C	6.012761000	2.770091000	-0.985869000
C	-2.928765000	-0.087240000	3.389342000
C	3.986980000	2.273323000	-2.416852000
B	1.207971000	0.664591000	-0.833712000
C	-4.994789000	-1.143009000	2.464205000
H	1.941653000	-3.117754000	-0.259121000
H	1.986948000	-4.088799000	1.233196000
H	3.272053000	-4.259455000	0.017826000
H	2.661278000	-1.759353000	1.684853000
H	4.945373000	-3.804097000	1.933442000
H	3.635297000	-3.526282000	3.102642000
H	4.886652000	-2.282214000	2.839483000
H	5.323081000	-3.329491000	-0.406830000
H	6.704120000	-2.085488000	-2.046138000
H	6.383627000	0.356634000	-2.327488000
H	4.027717000	2.409909000	-0.287276000
H	6.698607000	2.488793000	-1.800221000
H	6.531331000	2.576850000	-0.034176000
H	5.837891000	3.854965000	-1.065899000
H	4.606434000	1.921250000	-3.257319000
H	3.807220000	3.351458000	-2.557962000
H	3.018892000	1.756725000	-2.475471000
H	1.878823000	-0.173802000	3.211712000
H	2.995101000	0.602164000	4.360145000
H	3.502629000	-0.842252000	3.469125000
H	5.432611000	1.818059000	1.718452000
H	5.499926000	0.170172000	2.383116000
H	5.204272000	1.554741000	3.463825000
H	2.466075000	2.666694000	3.320260000
H	3.477750000	3.140357000	1.945317000
H	-0.524208000	1.732782000	1.308523000
H	-0.057399000	2.744708000	2.700807000
H	0.368454000	1.017566000	2.661311000
H	0.476721000	3.683864000	0.056194000

H	2.244130000	3.882421000	-0.014695000
H	1.338994000	4.427211000	1.419838000
H	1.067479000	3.204186000	-2.143154000
H	-0.813271000	4.148882000	-3.440506000
H	-2.988525000	2.918843000	-3.551420000
H	-3.261371000	0.788608000	-2.354695000
H	-1.987161000	4.088580000	1.233244000
H	-3.271516000	4.259148000	0.017074000
H	-1.941217000	3.117052000	-0.258735000
H	-3.636676000	3.526941000	3.101976000
H	-4.888333000	2.283280000	2.838322000
H	-4.945895000	3.804947000	1.931861000
H	-2.662282000	1.759414000	1.685225000
H	-5.323477000	3.329407000	-0.407339000
H	-6.704216000	2.085302000	-2.046819000
H	-6.383458000	-0.356784000	-2.328174000
H	-3.018988000	-1.756383000	-2.476427000
H	-4.606669000	-1.921263000	-3.257912000
H	-3.806990000	-3.351288000	-2.558708000
H	-6.530725000	-2.577159000	-0.034344000
H	-5.837263000	-3.855171000	-1.066180000
H	-6.698397000	-2.489187000	-1.800357000
H	-4.027201000	-2.409763000	-0.287982000
H	-3.502618000	0.841834000	3.468958000
H	-1.878869000	0.173313000	3.211391000
H	-2.995207000	-0.602817000	4.359652000
H	-5.499996000	-0.170163000	2.382656000
H	-5.204495000	-1.554972000	3.463099000
H	-5.432815000	-1.817924000	1.717667000
H	-3.478060000	-3.140444000	1.944319000
H	-2.466448000	-2.667139000	3.319436000
H	-2.244295000	-3.882268000	-0.015692000
H	-1.339346000	-4.427351000	1.418849000
H	-0.476880000	-3.683846000	0.055413000
H	-0.368689000	-1.017964000	2.660940000
H	0.524031000	-1.732960000	1.308077000
H	0.057106000	-2.745128000	2.700148000
H	3.261969000	-0.788425000	-2.353581000
H	2.989546000	-2.918598000	-3.550528000
H	0.814245000	-4.148630000	-3.440472000
H	-1.066970000	-3.204007000	-2.143741000

4b (closed-shell singlet), B3LYP-D3(BJ)/def2-SVP

E+ZPE = –2413.764114 E_h

Lowest frequency = 19.73 cm^{-1}

C	0.049617000	-1.472294000	-1.713721000
C	4.239768000	-1.607303000	-0.152095000
C	3.974054000	-0.221598000	-0.199841000
C	-0.100442000	2.961462000	3.085423000

H	-1.078597000	2.744510000	3.546142000
H	0.632270000	2.930162000	3.910527000
C	0.231046000	1.866592000	2.065465000
H	-0.578258000	1.768027000	1.326584000
H	0.296819000	0.899619000	2.576436000
C	-2.272925000	1.197198000	-2.660265000
H	-3.202774000	0.639653000	-2.769346000
C	1.540268000	2.132040000	1.283900000
B	-1.081683000	-0.615284000	-0.976946000
C	-3.974011000	0.221819000	-0.199764000
C	-0.230917000	-1.867162000	2.064890000
H	0.578185000	-1.768673000	1.325775000
H	-0.296355000	-0.900221000	2.575971000
C	0.100689000	-2.962206000	3.084622000
H	1.079067000	-2.745521000	3.545000000
H	-0.631717000	-2.930822000	3.909998000
C	0.111304000	-4.362002000	2.465406000
H	0.994905000	-4.454290000	1.817099000
H	0.220199000	-5.127437000	3.251680000
C	-1.138707000	-4.628700000	1.622578000
H	-2.020109000	-4.731468000	2.279593000
H	-1.038085000	-5.592415000	1.096269000
C	-1.369953000	-3.511799000	0.598104000
H	-2.252414000	-3.738270000	-0.019756000
H	-0.511521000	-3.464710000	-0.083115000
C	1.137945000	4.628367000	1.622947000
H	2.019586000	4.731420000	2.279598000
H	1.036797000	5.592049000	1.096680000
C	-4.239452000	1.607585000	-0.152144000
C	-5.258521000	2.124419000	-0.963630000
H	-5.467940000	3.195240000	-0.934530000
C	-5.989192000	1.310744000	-1.821285000
H	-6.774452000	1.736446000	-2.450365000
C	-5.703038000	-0.050486000	-1.879051000
H	-6.267361000	-0.689549000	-2.560975000
C	-4.708366000	-0.620266000	-1.076143000
C	-0.049659000	1.472067000	-1.713747000
C	-1.261841000	0.743729000	-1.805893000
C	1.867596000	1.005582000	0.284159000
B	1.081586000	0.615095000	-0.976848000
N	3.021937000	0.386817000	0.690074000
C	1.369167000	3.511545000	0.598377000
H	2.251379000	3.738280000	-0.019752000
H	0.510548000	3.464218000	-0.082594000
C	-0.111627000	4.361301000	2.466325000
H	-0.220329000	5.126659000	3.252702000
H	-0.995565000	4.453443000	1.818457000
C	-2.108050000	2.378129000	-3.396710000
H	-2.908164000	2.720494000	-4.058493000
C	-4.450075000	-2.112885000	-1.215864000
H	-3.748285000	-2.408702000	-0.430626000

C	-2.786072000	-2.152363000	2.204421000
H	-3.490422000	-2.920607000	1.849725000
H	-2.553996000	-2.391688000	3.249457000
C	-3.448592000	-0.775997000	2.078271000
C	-1.540443000	-2.132235000	1.283712000
C	-3.449075000	2.582395000	0.704957000
H	-2.654634000	2.013552000	1.204095000
C	4.708162000	0.620565000	-1.076340000
C	0.094426000	2.664684000	-2.436481000
H	1.020172000	3.240395000	-2.372184000
C	-0.094392000	-2.664918000	-2.436458000
H	-1.020173000	-3.240591000	-2.372304000
C	0.932665000	-3.119835000	-3.275284000
H	0.806189000	-4.047298000	-3.840638000
C	2.108158000	-2.378386000	-3.396493000
H	2.908340000	-2.720749000	-4.058193000
C	2.272944000	-1.197421000	-2.660080000
H	3.202765000	-0.639843000	-2.769180000
C	1.261812000	-0.743946000	-1.805766000
C	-0.932547000	3.119564000	-3.275428000
H	-0.806018000	4.047005000	-3.840807000
C	3.448750000	0.776154000	2.078178000
C	-3.781238000	-2.421551000	-2.563991000
H	-2.862190000	-1.835777000	-2.699090000
H	-4.455963000	-2.185588000	-3.402456000
H	-3.519551000	-3.489749000	-2.632992000
C	3.449586000	-2.582126000	0.705174000
H	2.655229000	-2.013300000	1.204453000
C	4.449577000	2.113124000	-1.216217000
H	3.747506000	2.408850000	-0.431194000
C	-2.888396000	0.200611000	3.126091000
H	-1.817092000	0.369117000	2.978723000
H	-3.036667000	-0.218507000	4.133102000
H	-3.395514000	1.170763000	3.089881000
C	-4.334263000	3.232427000	1.781007000
H	-3.721907000	3.828130000	2.477164000
H	-4.891954000	2.486671000	2.367299000
H	-5.074633000	3.910197000	1.326783000
C	-4.967081000	-0.855984000	2.226016000
H	-5.205734000	-1.213802000	3.239106000
H	-5.410475000	-1.552759000	1.503986000
H	-5.443319000	0.124521000	2.088083000
C	5.702917000	0.050898000	-1.879233000
H	6.267083000	0.690005000	-2.561246000
C	5.258893000	-2.124020000	-0.963578000
H	5.468525000	-3.194798000	-0.934387000
C	-5.718275000	-2.958688000	-1.029578000
H	-6.218121000	-2.741140000	-0.073269000
H	-5.464924000	-4.030891000	-1.044821000
H	-6.450061000	-2.783984000	-1.833673000
C	-2.773793000	3.659736000	-0.156868000

H	-2.314169000	4.427880000	0.481109000
H	-3.499651000	4.171303000	-0.806032000
H	-1.996442000	3.230644000	-0.797696000
C	2.774143000	-3.659520000	-0.156473000
H	3.499791000	-4.170774000	-0.806114000
H	1.996329000	-3.230552000	-0.796835000
H	2.315091000	-4.427883000	0.481656000
C	5.989357000	-1.310268000	-1.821343000
H	6.774687000	-1.735861000	-2.450411000
C	4.334981000	-3.232095000	1.781095000
H	4.892771000	-2.486295000	2.367241000
H	5.075274000	-3.909894000	1.326789000
H	3.722750000	-3.827755000	2.477398000
C	3.781011000	2.421570000	-2.564532000
H	3.519168000	3.489719000	-2.633669000
H	2.862084000	1.835642000	-2.699796000
H	4.455966000	2.185672000	-3.402829000
C	5.717561000	2.959192000	-1.029662000
H	5.464029000	4.031348000	-1.045185000
H	6.449646000	2.784450000	-1.833477000
H	6.217125000	2.741916000	-0.073145000
C	-1.867712000	-1.005702000	0.284060000
N	-3.021926000	-0.386771000	0.690091000
C	2.786122000	2.152462000	2.204270000
H	3.490302000	2.920676000	1.849160000
H	2.554355000	2.392013000	3.249320000
C	2.888880000	-0.200541000	3.126112000
H	3.396533000	-1.170427000	3.090133000
H	1.817692000	-0.369685000	2.978635000
H	3.036792000	0.218820000	4.133074000
C	4.967249000	0.856348000	2.225723000
H	5.410454000	1.553169000	1.503622000
H	5.443607000	-0.124091000	2.087751000
H	5.205968000	1.214219000	3.238779000

4b (open-shell singlet), UB3LYP-D3(BJ)/def2-SVP

E+ZPE = –2413.783645 E_h

Lowest frequency = 18.77 cm^{-1}

C	-0.015182000	-1.460693000	-1.625820000
C	4.300460000	-1.700623000	-0.147363000
C	4.105187000	-0.302632000	-0.212805000
C	0.056730000	2.999983000	2.965504000
H	-0.947968000	2.805704000	3.377205000
H	0.751382000	2.922892000	3.820202000
C	0.397402000	1.924275000	1.927851000
H	-0.387556000	1.884740000	1.155757000
H	0.403508000	0.936071000	2.405671000
C	-2.243985000	1.314442000	-2.530312000
H	-3.191656000	0.790132000	-2.645055000

C	1.749198000	2.173721000	1.209112000
B	-1.221246000	-0.638008000	-0.996930000
C	-4.105246000	0.302589000	-0.212737000
C	-0.397514000	-1.923988000	1.928037000
H	0.387526000	-1.884305000	1.156033000
H	-0.403891000	-0.935770000	2.405824000
C	-0.056736000	-2.999588000	2.965765000
H	0.947875000	-2.805088000	3.377574000
H	-0.751499000	-2.922605000	3.820382000
C	-0.119447000	-4.415271000	2.384054000
H	0.723231000	-4.548719000	1.689393000
H	0.012072000	-5.163463000	3.183496000
C	-1.419102000	-4.668458000	1.615423000
H	-2.269856000	-4.716196000	2.317404000
H	-1.376623000	-5.653404000	1.121420000
C	-1.657648000	-3.578091000	0.564538000
H	-2.574702000	-3.789069000	-0.008127000
H	-0.825812000	-3.589508000	-0.150030000
C	1.419617000	4.668521000	1.615289000
H	2.270288000	4.716087000	2.317383000
H	1.377424000	5.653465000	1.121258000
C	-4.300486000	1.700585000	-0.147238000
C	-5.266074000	2.283110000	-0.979182000
H	-5.422648000	3.362525000	-0.935905000
C	-6.006994000	1.523395000	-1.877305000
H	-6.745997000	2.000838000	-2.525099000
C	-5.794029000	0.149079000	-1.948321000
H	-6.370821000	-0.448056000	-2.657428000
C	-4.860402000	-0.485707000	-1.120908000
C	0.015096000	1.460686000	-1.625774000
C	-1.236351000	0.778490000	-1.715644000
C	2.069537000	1.050789000	0.203755000
B	1.221176000	0.637977000	-0.996934000
N	3.178593000	0.360382000	0.657460000
C	1.658055000	3.578071000	0.564465000
H	2.575220000	3.788827000	-0.008103000
H	0.826302000	3.589647000	-0.150196000
C	0.119804000	4.415633000	2.383750000
H	-0.011680000	5.163879000	3.183147000
H	-0.722745000	4.549223000	1.688959000
C	-2.050030000	2.504349000	-3.240163000
H	-2.847909000	2.889903000	-3.880133000
C	-4.688466000	-1.991471000	-1.258196000
H	-4.029635000	-2.328587000	-0.452169000
C	-2.961285000	-2.130679000	2.171262000
H	-3.708579000	-2.870065000	1.843706000
H	-2.706192000	-2.375098000	3.209817000
C	-3.570607000	-0.728579000	2.057959000
C	-1.749167000	-2.173744000	1.209137000
C	-3.496627000	2.614819000	0.763506000
H	-2.728364000	2.002228000	1.250915000

C	4.860336000	0.485725000	-1.120932000
C	0.175828000	2.673054000	-2.317841000
H	1.131203000	3.199764000	-2.273514000
C	-0.175936000	-2.673035000	-2.317931000
H	-1.131310000	-3.199746000	-2.273596000
C	0.842903000	-3.193354000	-3.124679000
H	0.683154000	-4.125168000	-3.673839000
C	2.049912000	-2.504311000	-3.240277000
H	2.847779000	-2.889851000	-3.880271000
C	2.243891000	-1.314439000	-2.530375000
H	3.191573000	-0.790141000	-2.645093000
C	1.236265000	-0.778501000	-1.715689000
C	-0.843029000	3.193401000	-3.124548000
H	-0.683295000	4.125239000	-3.673673000
C	3.570535000	0.728367000	2.057957000
C	-3.998729000	-2.344249000	-2.584706000
H	-3.041990000	-1.813901000	-2.689680000
H	-4.630041000	-2.070683000	-3.445357000
H	-3.797736000	-3.426241000	-2.644159000
C	3.496668000	-2.614903000	0.763400000
H	2.728500000	-2.002312000	1.250962000
C	4.688367000	1.991493000	-1.258138000
H	4.029386000	2.328526000	-0.452197000
C	-2.934721000	0.220207000	3.091281000
H	-1.869955000	0.377674000	2.883578000
H	-3.028116000	-0.218034000	4.096744000
H	-3.427770000	1.197696000	3.108919000
C	-4.390329000	3.239606000	1.848475000
H	-3.780464000	3.787494000	2.584967000
H	-4.981517000	2.484257000	2.387150000
H	-5.101512000	3.955991000	1.406741000
C	-5.086506000	-0.750483000	2.249264000
H	-5.314833000	-1.094038000	3.269697000
H	-5.572714000	-1.433690000	1.540609000
H	-5.529609000	0.246153000	2.116771000
C	5.793976000	-0.149004000	-1.948374000
H	6.370756000	0.448175000	-2.657454000
C	5.266059000	-2.283090000	-0.979337000
H	5.422672000	-3.362500000	-0.936092000
C	-6.013254000	-2.753185000	-1.106755000
H	-6.519812000	-2.501680000	-0.162126000
H	-5.830883000	-3.839846000	-1.116914000
H	-6.712450000	-2.530517000	-1.927946000
C	-2.768826000	3.716266000	-0.020703000
H	-2.327812000	4.443781000	0.675588000
H	-3.454977000	4.272259000	-0.676704000
H	-1.968469000	3.304903000	-0.645132000
C	2.768706000	-3.716239000	-0.020818000
H	3.454735000	-4.272202000	-0.676968000
H	1.968284000	-3.304774000	-0.645094000
H	2.327746000	-4.443804000	0.675456000

C	6.006961000	-1.523321000	-1.877427000
H	6.745978000	-2.000719000	-2.525239000
C	4.390474000	-3.239832000	1.848201000
H	4.981791000	-2.484565000	2.386849000
H	5.101545000	-3.956237000	1.406319000
H	3.780670000	-3.787728000	2.584737000
C	3.998859000	2.344343000	-2.584747000
H	3.797824000	3.426331000	-2.644154000
H	3.042167000	1.813957000	-2.689941000
H	4.630346000	2.070873000	-3.445300000
C	6.013096000	2.753250000	-1.106407000
H	5.830674000	3.839903000	-1.116470000
H	6.712431000	2.530714000	-1.927514000
H	6.519517000	2.501659000	-0.161727000
C	-2.069610000	-1.050881000	0.203734000
N	-3.178654000	-0.360485000	0.657489000
C	2.961197000	2.130455000	2.171388000
H	3.708545000	2.869877000	1.844043000
H	2.705979000	2.374725000	3.209948000
C	2.934668000	-0.220535000	3.091182000
H	3.427718000	-1.198025000	3.108700000
H	1.869903000	-0.377982000	2.883472000
H	3.028077000	0.217592000	4.096692000
C	5.086436000	0.750271000	2.249256000
H	5.572633000	1.433537000	1.540649000
H	5.529546000	-0.246351000	2.116682000
H	5.314767000	1.093749000	3.269713000

4b (triplet), UB3LYP-D3(BJ)/def2-SVP

E+ZPE = –2413.783412 E_h

Lowest frequency = 18.39 cm^{-1}

C	-0.018387000	-1.460166000	-1.620792000
C	4.304258000	-1.704138000	-0.145968000
C	4.111450000	-0.305837000	-0.213856000
C	0.065420000	3.000743000	2.961052000
H	-0.939830000	2.806555000	3.371485000
H	0.759164000	2.922383000	3.816377000
C	0.406523000	1.926161000	1.922347000
H	-0.377913000	1.888681000	1.149592000
H	0.411206000	0.937107000	2.398637000
C	-2.242803000	1.320982000	-2.522062000
H	-3.191233000	0.798273000	-2.637271000
C	1.759202000	2.176050000	1.205065000
B	-1.226850000	-0.638260000	-0.997539000
C	-4.111452000	0.305807000	-0.213893000
C	-0.406403000	-1.926266000	1.922217000
H	0.377979000	-1.888810000	1.149408000
H	-0.410980000	-0.937232000	2.398554000
C	-0.065295000	-3.000917000	2.960851000

H	0.940009000	-2.806820000	3.371199000
H	-0.758956000	-2.922538000	3.816241000
C	-0.129832000	-4.417144000	2.380542000
H	0.711503000	-4.551639000	1.684406000
H	0.002724000	-5.164676000	3.180436000
C	-1.430911000	-4.670374000	1.614330000
H	-2.280674000	-4.716382000	2.317614000
H	-1.389974000	-5.656065000	1.121685000
C	-1.669702000	-3.581243000	0.562217000
H	-2.587608000	-3.791970000	-0.009234000
H	-0.838521000	-3.594725000	-0.152994000
C	1.430765000	4.670357000	1.614433000
H	2.280596000	4.716441000	2.317630000
H	1.389679000	5.656051000	1.121807000
C	-4.304284000	1.704105000	-0.146045000
C	-5.267327000	2.289927000	-0.978600000
H	-5.422057000	3.369536000	-0.933565000
C	-6.007921000	1.533251000	-1.879581000
H	-6.744777000	2.013289000	-2.527904000
C	-5.797557000	0.158627000	-1.952638000
H	-6.374319000	-0.436115000	-2.663782000
C	-4.866703000	-0.479476000	-1.124641000
C	0.018470000	1.460262000	-1.620778000
C	-1.234966000	0.780381000	-1.710149000
C	2.079543000	1.053280000	0.199386000
B	1.226948000	0.638353000	-0.997568000
N	3.186470000	0.360377000	0.655336000
C	1.669565000	3.581268000	0.562277000
H	2.587405000	3.792090000	-0.009247000
H	0.838323000	3.594684000	-0.152866000
C	0.129790000	4.416994000	2.380784000
H	-0.002733000	5.164492000	3.180714000
H	-0.711639000	4.551444000	1.684753000
C	-2.047661000	2.511428000	-3.230083000
H	-2.845480000	2.899224000	-3.868761000
C	-4.698124000	-1.985518000	-1.263492000
H	-4.040936000	-2.325089000	-0.457117000
C	-2.970371000	-2.131383000	2.168108000
H	-3.718996000	-2.869458000	1.840619000
H	-2.714942000	-2.376760000	3.206345000
C	-3.577631000	-0.728367000	2.055904000
C	-1.759155000	-2.176022000	1.205035000
C	-3.500682000	2.615093000	0.768224000
H	-2.733819000	2.000395000	1.255116000
C	4.866639000	0.479396000	-1.124693000
C	0.179628000	2.674176000	-2.310695000
H	1.136455000	3.198295000	-2.267945000
C	-0.179545000	-2.674088000	-2.310693000
H	-1.136370000	-3.198209000	-2.267928000
C	0.839102000	-3.197868000	-3.114946000
H	0.677963000	-4.130003000	-3.663150000

C	2.047711000	-2.511319000	-3.230155000
H	2.845516000	-2.899105000	-3.868855000
C	2.242852000	-1.320857000	-2.522161000
H	3.191261000	-0.798126000	-2.637427000
C	1.235043000	-0.780271000	-1.710204000
C	-0.839032000	3.197954000	-3.114931000
H	-0.677898000	4.130079000	-3.663153000
C	3.577730000	0.728467000	2.055905000
C	-4.007805000	-2.338735000	-2.589577000
H	-3.049417000	-1.810939000	-2.692586000
H	-4.637246000	-2.062478000	-3.450744000
H	-3.809733000	-3.421220000	-2.649953000
C	3.500642000	-2.615086000	0.768327000
H	2.733732000	-2.000385000	1.255136000
C	4.698035000	1.985428000	-1.263630000
H	4.040937000	2.325053000	-0.457203000
C	-2.939513000	0.218577000	3.089714000
H	-1.874992000	0.376090000	2.880404000
H	-3.031174000	-0.221446000	4.094556000
H	-3.432204000	1.196162000	3.110198000
C	-4.395588000	3.237898000	1.853386000
H	-3.786471000	3.783236000	2.592385000
H	-4.988516000	2.481784000	2.389028000
H	-5.105255000	3.956164000	1.412261000
C	-5.093469000	-0.748333000	2.248077000
H	-5.321859000	-1.092047000	3.268443000
H	-5.580809000	-1.430598000	1.539244000
H	-5.535369000	0.248907000	2.116185000
C	5.797432000	-0.158756000	-1.952723000
H	6.374147000	0.435945000	-2.663938000
C	5.267239000	-2.290010000	-0.978556000
H	5.421942000	-3.369623000	-0.933500000
C	-6.025024000	-2.743959000	-1.114068000
H	-6.531659000	-2.492123000	-0.169558000
H	-5.845506000	-3.831085000	-1.125269000
H	-6.722969000	-2.518531000	-1.935573000
C	-2.770200000	3.718168000	-0.011154000
H	-2.329718000	4.442742000	0.688560000
H	-3.454403000	4.277229000	-0.666605000
H	-1.969081000	3.308006000	-0.635415000
C	2.770237000	-3.718248000	-0.011002000
H	3.454476000	-4.277264000	-0.666453000
H	1.969064000	-3.308178000	-0.635258000
H	2.329842000	-4.442835000	0.688755000
C	6.007789000	-1.533379000	-1.879613000
H	6.744594000	-2.013453000	-2.527967000
C	4.395512000	-3.237774000	1.853585000
H	4.988369000	-2.481592000	2.389212000
H	5.105242000	-3.956038000	1.412558000
H	3.786377000	-3.783091000	2.592584000
C	4.007542000	2.338543000	-2.589651000

H	3.809455000	3.421022000	-2.650080000
H	3.049142000	1.810734000	-2.692493000
H	4.636869000	2.062229000	-3.450882000
C	6.024951000	2.743885000	-1.114425000
H	5.845433000	3.831011000	-1.125711000
H	6.722804000	2.518374000	-1.935985000
H	6.531692000	2.492141000	-0.169948000
C	-2.079478000	-1.053231000	0.199374000
N	-3.186435000	-0.360359000	0.655300000
C	2.970493000	2.131495000	2.168037000
H	3.719095000	2.869531000	1.840400000
H	2.715166000	2.376976000	3.206274000
C	2.939665000	-0.218402000	3.089823000
H	3.432452000	-1.195935000	3.110450000
H	1.875163000	-0.376050000	2.880514000
H	3.031261000	0.221762000	4.094610000
C	5.093576000	0.748438000	2.248019000
H	5.580891000	1.430660000	1.539128000
H	5.535467000	-0.248810000	2.116171000
H	5.322003000	1.092213000	3.268357000

5b, B3LYP-D3(BJ)/def2-SVP
E+ZPE = –2527.034075 E_h
Lowest frequency = 20.82 cm^{-1}

O	-0.490282000	0.494803000	-1.785501000
C	-0.280042000	0.423650000	-0.596954000
N	-3.501967000	-0.203462000	-0.635167000
C	-2.733501000	0.556847000	0.155820000
C	-4.369242000	0.575318000	-1.599847000
C	-4.471586000	1.916080000	-0.869386000
H	-5.384482000	1.929043000	-0.254462000
C	-3.224074000	2.018339000	0.042615000
C	-3.653037000	0.753274000	-2.948590000
H	-4.200469000	1.497322000	-3.547932000
H	-2.619384000	1.089690000	-2.803178000
C	-5.728733000	-0.085176000	-1.817532000
H	-6.328194000	0.552406000	-2.484858000
H	-6.282555000	-0.208920000	-0.877727000
H	-5.628040000	-1.072757000	-2.289137000
C	-3.646631000	2.571588000	1.428023000
H	-2.780487000	2.549097000	2.094665000
H	-4.414028000	1.920022000	1.873422000
H	-5.055719000	4.077382000	0.716453000
H	-4.449797000	4.353186000	2.350819000
C	-3.065425000	4.935613000	0.777113000
C	-2.583762000	4.430572000	-0.584339000
H	-3.392031000	4.551722000	-1.327418000
C	2.503419000	0.779341000	-0.301455000
N	3.745722000	0.354144000	-0.137932000

C	4.834770000	1.373399000	-0.466799000
C	3.992312000	2.615883000	-0.788640000
H	4.410322000	3.171171000	-1.638034000
H	3.989538000	3.297520000	0.074923000
C	2.555141000	2.121662000	-1.045363000
C	5.727092000	0.938458000	-1.636161000
H	6.536589000	1.675443000	-1.741406000
H	5.190110000	0.901141000	-2.590571000
H	6.187349000	-0.041162000	-1.448539000
C	5.729909000	1.599699000	0.752589000
H	6.453607000	2.392727000	0.512121000
H	6.293496000	0.693903000	1.014179000
H	5.152217000	1.923127000	1.627204000
C	2.312312000	1.767750000	-2.549665000
H	3.097427000	1.087407000	-2.905555000
H	1.360963000	1.221306000	-2.620046000
C	2.262135000	3.001198000	-3.456602000
H	3.255753000	3.483123000	-3.501861000
H	2.034319000	2.674052000	-4.484472000
C	1.231509000	4.021914000	-2.974370000
H	1.236329000	4.912493000	-3.624355000
H	0.222211000	3.579328000	-3.044439000
C	1.507698000	4.406198000	-1.520973000
H	0.743847000	5.111748000	-1.155387000
H	2.473744000	4.939236000	-1.455415000
C	1.516074000	3.172623000	-0.615683000
H	0.523005000	2.717378000	-0.644798000
H	1.690171000	3.461505000	0.427810000
C	-3.732761000	-1.613094000	-0.427427000
C	-3.180172000	-2.595575000	-1.271644000
C	-3.496077000	-3.938068000	-1.018241000
H	-3.061477000	-4.711901000	-1.653310000
C	-4.328957000	-4.303997000	0.032212000
H	-4.553884000	-5.357579000	0.214457000
C	-4.870484000	-3.320147000	0.856419000
H	-5.517383000	-3.610553000	1.685714000
C	-4.591144000	-1.965563000	0.645347000
C	-2.244108000	-2.279210000	-2.423516000
H	-2.032441000	-1.204750000	-2.401334000
C	-5.173624000	-0.938071000	1.607697000
H	-5.109916000	0.044704000	1.127350000
C	-0.895380000	-2.995509000	-2.278724000
H	-0.229956000	-2.697105000	-3.102081000
H	-1.007582000	-4.090686000	-2.321594000
H	-0.407689000	-2.741823000	-1.330775000
C	-2.892476000	-2.635821000	-3.771866000
H	-2.264298000	-2.286362000	-4.607025000
H	-3.892132000	-2.189457000	-3.885738000
H	-3.010536000	-3.726509000	-3.876960000
C	-4.344894000	-0.863447000	2.900748000
H	-4.747399000	-0.090804000	3.575785000

H	-3.294548000	-0.622067000	2.689875000
H	-4.366989000	-1.826713000	3.434863000
C	-6.656638000	-1.180756000	1.919471000
H	-7.062238000	-0.339728000	2.504016000
H	-6.809414000	-2.093034000	2.516793000
H	-7.254455000	-1.279444000	0.999890000
C	4.155485000	-0.991388000	0.219668000
C	4.312256000	-1.923623000	-0.836361000
C	4.789814000	-3.201844000	-0.525144000
H	4.906786000	-3.935884000	-1.323089000
C	5.091292000	-3.559910000	0.786278000
H	5.459530000	-4.563782000	1.010109000
C	4.892727000	-2.643740000	1.812232000
H	5.088750000	-2.941470000	2.843417000
C	4.416461000	-1.348495000	1.561031000
C	3.883096000	-1.629738000	-2.266520000
H	3.854183000	-0.546351000	-2.389483000
C	4.117303000	-0.458307000	2.755738000
H	3.833903000	0.525809000	2.375355000
C	4.857507000	-2.173755000	-3.318019000
H	4.577299000	-1.802480000	-4.316335000
H	4.838309000	-3.273599000	-3.367953000
H	5.894511000	-1.863685000	-3.116165000
C	2.453197000	-2.125501000	-2.522407000
H	2.156241000	-1.906596000	-3.560472000
H	1.731562000	-1.640889000	-1.854436000
H	2.370784000	-3.211410000	-2.360918000
C	5.327936000	-0.277765000	3.683632000
H	5.092476000	0.459097000	4.467677000
H	6.221753000	0.070288000	3.145411000
H	5.590629000	-1.218975000	4.191790000
C	2.911124000	-0.991408000	3.547309000
H	2.664863000	-0.299025000	4.366343000
H	3.130653000	-1.980278000	3.981090000
H	2.023577000	-1.087647000	2.911553000
C	0.678450000	-1.345237000	0.678513000
B	1.126079000	0.191867000	0.287222000
C	1.377588000	-2.557220000	0.762304000
H	2.430239000	-2.610069000	0.517261000
B	-1.355654000	0.131061000	0.780775000
C	0.752712000	-3.739577000	1.174777000
H	1.336171000	-4.662430000	1.239127000
C	-0.599110000	-3.730072000	1.515999000
H	-1.093623000	-4.643241000	1.858643000
C	-1.329758000	-2.543884000	1.401358000
H	-2.387157000	-2.562047000	1.654661000
C	-0.728340000	-1.358549000	0.961524000
C	-0.598590000	1.008784000	1.904074000
C	-1.043262000	1.494257000	3.142815000
H	-2.072839000	1.316455000	3.462365000
C	-0.190000000	2.195885000	4.004679000

H	-0.566553000	2.572484000	4.959856000
C	1.138503000	2.415902000	3.636611000
H	1.806025000	2.989155000	4.286103000
C	1.619821000	1.862849000	2.443467000
H	2.671393000	2.018774000	2.188423000
C	0.785805000	1.123259000	1.599940000
H	-4.553215000	2.749769000	-1.577897000
H	-3.627002000	-0.179545000	-3.520899000
C	-4.149305000	4.016062000	1.344972000
H	-3.434053000	5.971934000	0.698494000
H	-2.211163000	4.953480000	1.477345000
H	-1.744116000	5.045648000	-0.948441000
C	-2.138849000	2.967123000	-0.522234000
H	-1.268397000	2.893073000	0.143888000
H	-1.812048000	2.625285000	-1.512124000

S5 Additional References

[1] S. Stoll, A. Schweiger, *J. Magn. Reson.* **2006**, *178*, 42.

[2] V. Lavallo, Y. Canac, C. Präsang, B. Donnadieu, G. Bertrand, *Ange. Chem. Int. Ed.* **2005**, *44*, 5705.

[3] S. Bieller, F. Zhang, M. Bolte, J. W. Bats, H.-W. Lerner, M. Wagner, *Organometallics* **2004**, *23*, 2107.

[4] B. Bogdanović, K. Schlichte, U. Westeppe, *Chem. Ber.* **1988**, *121*, 27.

[5] G. Sheldrick, *Acta Cryst.* **2015**, *A71*, 3.

[6] G. Sheldrick, *Acta Cryst.* **2008**, *A64*, 112.

[7] a) S. H. Vosko, L. Wilk, M. Nusair, *Can. J. Phys.* **1980**, *58*, 1200; b) C. Lee, W. Yang, R. G. Parr, *Phys. Rev. B.* **1988**, *37*, 785; c) A. D. Becke, *J. Chem. Phys.* **1993**, *98*, 5648; d) P. J. Stephens, F. J. Devlin, C. F. Chabalowski, M. J. Frisch, *J. Phys. Chem.* **1994**, *98*, 11623.

[8] S. Grimme, J. Antony, S. Ehrlich, H. Krieg, *J. Chem. Phys.* **2010**, *132*, 154104.

[9] S. Grimme, S. Ehrlich, L. Goerigk, *J. Comput. Chem.* **2011**, *32*, 1456.

[10] F. Weigend, R. Ahlrichs, *Phys. Chem. Chem. Phys.* **2005**, *7*, 3297.

[11] J. Michl, V. Bonačić-Koutecký, *Tetrahedron* **1988**, *44*, 7559.

[12] B. O. Roos, in Advances in Chemical Physics, Wiley, Hoboken, New Jersey, 1987, 399.

[13] a) C. Angeli, R. Cimiraglia, S. Evangelisti, T. Leininger, J.-P. Malrieu, *J. Chem. Phys.* **2001**, *114*, 10252; b) C. Angeli, R. Cimiraglia, J.-P. Malrieu, *Chem. Phys. Lett.* **2001**, *350*, 297; c) C. Angeli, R. Cimiraglia, J.-P. Malrieu, *J. Chem. Phys.* **2002**, *117*, 9138.

[14] a) E. J. Baerends, D. E. Ellis, P. Ros, *Chem. Phys.* **1973**, *2*, 41; b) J. L. Whitten, *J. Chem. Phys.* **1973**, *58*, 4496; c) B. I. Dunlap, J. W. D. Connolly, J. R. Sabin, *J. Chem. Phys.* **1979**, *71*, 3396; d) C. Van Alsenoy, *J. Comput. Chem.* **1988**, *9*, 620; e) K. Eichkorn, O. Treutler, H. Öhm, M. Häser, R. Ahlrichs, *Chem. Phys. Lett.* **1995**, *240*, 283; f) R. A. Kendall, H. A. Früchtl, *Theor. Chem. Acc.* **1997**, *97*, 158; g) K. Eichkorn, F. Weigend, O. Treutler, R. Ahlrichs, *Theor. Chem. Acc.* **1997**, *97*, 119; h) F. Neese, *J. Comput. Chem.* **2003**, *24*, 1740.

[15] M. J. Frisch, G. W. Trucks, H. B. Schlegel, G. E. Scuseria, M. A. Robb, J. R. Cheeseman, G. Scalmani, V. Barone, B. Mennucci, G. A. Petersson, H. Nakatsuji, M. Caricato, X. Li, H. P. Hratchian, A. F. Izmaylov, J. Bloino, G. Zheng, J. L. Sonnenberg, M. Hada, M. Ehara, K. Toyota, R. Fukuda, J. Hasegawa, M. Ishida, T. Nakajima, Y. Honda, O. Kitao, H. Nakai, T. Vreven, J. A. Montgomery Jr., J. E. Peralta, F. Ogliaro, M. Bearpark, J. J. Heyd, E. Brothers, K. N. Kudin, V. N. Staroverov, R. Kobayashi, J. Normand, K. Raghavachari, A. Rendell, J. C. Burant, S. S. Iyengar, J. Tomasi, M. Cossi, N. Rega, J. M. Millam, M. Klene, J. E. Knox, J. B. Cross, V. Bakken, C. Adamo, J. Jaramillo, R. Gomperts, R. E. Stratmann, O. Yazyev, A. J. Austin, R. Cammi, C. Pomelli, J. W. Ochterski, R. L. Martin, K. Morokuma, V. G. Zakrzewski, G. A. Voth, P. Salvador, J. J. Dannenberg, S. Dapprich, A. D. Daniels, Ö. Farkas, J. B. Foresman, J. V. Ortiz, J. Cioslowski, D. J. Fox, Gaussian 16, Revision B.01.

[16] F. Neese, *WIREs Comput. Mol. Sci.* **2012**, *2*, 73.

[17] CYLview, 1.0b; C. Y. Legault, Université de Sherbrooke, 2009 (http://www.cylview.org).

[18] a) K. Yamaguchi, Chem. Phys. Lett. **1975**, *33*, 330; b) S. Yamanaka, M. Okumura, M. Nakano, K. Yamaguchi, *J. Mol. Struct.* **1994**, *310*, 205; c) M. Nakano, *Top. Curr. Chem.* **2017**, *375*, 47.

[19] D. Doehnert, J. Koutecky, *J. Am. Chem. Soc.* **1980**, *102*, 1789.
